# *cidalsDB*: an AI-empowered platform for anti-pathogen therapeutics research

**DOI:** 10.1186/s13321-024-00929-7

**Published:** 2024-11-28

**Authors:** Emna Harigua-Souiai, Ons Masmoudi, Samer Makni, Rafeh Oualha, Yosser Z. Abdelkrim, Sara Hamdi, Oussama Souiai, Ikram Guizani

**Affiliations:** 1 Laboratory of Molecular Epidemiology and Experimental Pathology - LR16IPT04, Institut Pasteur de Tunis, Université de Tunis El Manar, 13, Place Pasteur, 1002 Tunis, Tunisia; 2 Laboratory of BioInformatics, BioMathematics and BioStatistics - LR20IPT09, Institut Pasteur de Tunis, Université de Tunis El Manar, 13, Place Pasteur, 1002 Tunis, Tunisia; 3 Institut Supérieur des technologies médicales de Tunis, ISTMT, Université de Tunis El Manar, 9, Rue Docteur Zouheïr Safi, 1006 Tunis, Tunisia

**Keywords:** Machine learning, Deep learning, Drug discovery, Database

## Abstract

Computer-aided drug discovery (CADD) is nurtured by late advances in big data analytics and Artificial Intelligence (AI) towards enhanced drug discovery (DD) outcomes. In this context, reliable datasets are of utmost importance. We herein present *CidalsDB* a novel web server for AI-assisted DD against infectious pathogens, namely *Leishmania* parasites and Coronaviruses. We performed a literature search on molecules with validated anti-pathogen effects. Then, we consolidated these data with bioassays from PubChem. Finally, we constructed a database to store these datasets and make them accessible and ready-to-use for the scientific community through *CidalsDB*, a web-based interface. In a second step, we implemented and optimized four machine learning (ML) and three deep learning (DL) algorithms that optimally predicted the biological activity of molecules. Random Forests (RF), Multi-Layer Perceptron (MLP) and ChemBERTa were the best classifiers of anti-*Leishmania* molecules, while Gradient Boosting (GB), Graph-Convolutional Network (GCN) and ChemBERTa achieved the best performances on the Coronaviruses dataset. All six models were optimized and deployed through *CidalsDB* as anti-pathogen activity prediction models.

**Scientific contribution**

CidalsDB is an open access web-based tool that allows browsing and access to ready-to-use datasets of anti-pathogen molecules, alongside best performing AI models for biological activity prediction. It offers a democratized no-code platform for AI-based CADD, which shall foster innovation and collaboration within the DD community. *CidalsDB* is accessible through https://cidalsdb.streamlit.app/.

**Supplementary Information:**

The online version contains supplementary material available at 10.1186/s13321-024-00929-7.

## Introduction

Databases have marked a turning point in the history of biomedical research, providing opportunities for easy and seamless exploitation of data. As a result, several initiatives have been made to collect, organize and curate data related to genes, proteins, molecular targets and chemical compounds of biological interest. Key databases include NCBI [1], UNIPROT [2], Zinc database [3], ChemSpider [4], CheBI [5], PubChem [6, 7] and the RCSB PDB [8]. Recently, the Nobel prize in chemistry for the year 2024 was awarded to the Google DeepMind team that developed AlphaFold [9]. Such an achievement would never have been possible without the quality and quantity of data that was made accessible through the RCSB PDB database since 1971 [8]. This is the ultimate proof of the central role that data and databases play in leveraging Artificial Intelligence (AI) for scientific discoveries.

Noticeably, there is still disparity between curated databases of proteins and genes in comparison to those of chemical compounds. While repositories of gene and protein-related information are well-established and comprehensive, those focusing on chemical compounds are lagging behind. Databases dedicated to chemical information may exhibit variations in completeness and standardization, creating a gap in the accessibility and interoperability of their content. Closing this gap requires concerted efforts of compilation, curation, integration, and standardization of chemical compound repositories. Databases such as PubChem [7], ChemSpider [4] and CheBI [5] present valuable resources. They all contain large amounts of data on chemical entities of interest to diverse research questions and fields, including chemistry, bioinformatics, drug discovery, etc. In fact, ChemSpider is a comprehensive database of chemical structures, synthesis information, and chemical suppliers, that is ideal for chemists. ChEBI is a highly curated database, tailored to bioinformatics and researchers focused on small molecules in biological contexts. PubChem is more suited for researchers interested in bioactivity data, drug discovery, and biological assay results with less curation efforts than ChEBI. The bioassays section of PubChem is a particularly valuable resource for the Drug Discovery research.

Multiple chemical databases and data collections have been deployed with a focus on a particular disease, such as the Malaria Box, a collection of drug-like compounds designed to target *Plasmodium falciparum* [10], that was also exploited in Drug Discovery (DD) projects targeting other Neglected Tropical Diseases (NTDs) [11, 12]. Recently, a database called the LeishInDB was published [13]. It offers a browsing functionality through 8273 molecules with experimentally validated effects on *Leishmania* parasites issued from the literature. Other databases were conceived with larger scope such as the AntiPseudoBase focused on diverse antimicrobial agents [14], or the traditional Chinese Medicine bank (TCMBank) that gathers data on 9,191 herbs and 61,965 ingredients with described effects on 32,529 diseases [15], collected through an Ensemble Learning (EL) module and a series of chemical structure recognition tools.

Nonetheless, significant amounts of data occur in literature, and are not stored in databases. These can be found as free text and thus in unstructured formats. This constitutes a major challenge to automatic or high throughput retrieval, alongside the complexity of establishing the connections and dependencies between the chemical structure information and the molecule identifier in such formats (text format). The former mainly occurs as graphical representations of the molecule’s 2D structure, and the latter can occur as a common name, a chemical name, a brand name for a molecule, or just a number (e.g. compound 1, compound 2, etc) [16]. Extracting such data towards storage and archival in dedicated databases through automatic and reliable systems remains a challenge. Thus, manual extraction remains the most reliable way despite it being a fastidious task that requires expertise and dedication. Multiple research groups undertook such efforts towards gathering data sets of chemical entities of biological interest, with various research outcomes that ranges from collated datasets valuable for Computer-Aided Drug Discovery (CADD) projects [17–19] to databases accessible through web interfaces facilitating data browsing and partial retrieval upon filtering [13, 14].

The significance of collecting focused and diverse chemical libraries has been underscored by the SARS-CoV-2 pandemic, emphasizing the necessity for such structured data sources to develop successful streamlined and potent methods. Specifically, applications of computational approaches, including virtual screening and artificial intelligence (AI) offer the potential to evaluate billions of molecules as drug candidates. This not only broadens the scope of the search but also concurrently cuts costs and accelerates the drug discovery process [20]. Concrete efforts towards consolidating useful datasets for DD against COVID-19 led to collecting more than 54 billion molecules through the Joint European Disruptive Initiative (JEDI) grand challenge [21] and the MEDIATE platform [22]. More than 870 molecules were synthesized as part of the JEDI initiative for further testing. However, none have yet reached the final stages of development to reach the market. This highlights the complexity of the drug discovery process and the underlying challenges through the synthesis process, the clinical trials and the regulatory approvals.

Our group has contributed to these efforts through collecting a diverse dataset of anti-coronavirus molecules that successfully leveraged the power of machine learning (ML) and deep learning (DL) algorithms in predicting potential drug candidates [17]. Particularly, the Graph-Convolutional Network (GCN) model demonstrated satisfactory performances with high predictive power based on a True Positive Rate (TPR) equal to 62% and a True Negative Rate (TNR) equal to 95%, assessed through an external validation. Overall, a diverse, balanced and highly informative dataset proved to play a central role in successfully implementing ML and DL models for molecules’ activity prediction tasks [17]. In the absence of dedicated datasets, the PubChem bioassays appeared to be a valuable source of data. Through a repurposing strategy of drugs against Leishmaniasis, our group trained multiple ML models on a PubChem bioassay, and predicted 19 molecules as active compounds. Interestingly, 7 out of 19 compounds were described in the literature for being active against different parasite species of *Leishmania* [23], which validates the successful combination of the chosen dataset, the encoding system and the model training and optimization procedure [23]. In a succeeding research work, we have conducted the in vitro validation of ten out of the remaining predicted molecules on parasites growth. Five molecules proved to inhibit three different strains of *Leishmania* parasites at promising $\hbox {IC}_{{50}}$ values [24].

Despite the successful outcomes of these previous research projects [17, 23], multiple challenges towards optimal predictive models building were encountered and are worthy of being further investigated. These include data imbalance (few active molecules) in the DD field, multiplicity and differences in data encoding systems, and intrinsic characteristics of datasets that may drive biases in predictions. In the present work, we present a novel approach for data enhancement, through enrichment, that shall outperform synthetic data augmentation towards class balancing. We leveraged literature data to constitute valuable datasets of interest in the DD applications against *Leishmania* parasites and Coronaviruses as case studies.

For both pathogen groups, we collected diverse information on molecules described in the literature for their anti-pathogen effects that we consolidated with data issued from PubChem bioassays datasets. We trained ML and DL models able to predict the potential of an entry compound to be effective against the pathogen of interest. Throughout the optimization of these models, we assessed the impact of data imbalance on the models performances and demonstrated the usefulness of the literature data points as an enrichment strategy for large bioassay datasets. The literature-enriched datasets for both pathogen groups were deployed through a web interface, called *CidalsDB*, offering a chemical similarity-based browsing functionality and an activity prediction module based on the ML and DL models herein optimized. This approach allows the CADD community to explore and utilize datasets and predictive models focused on *Leishmania* parasites and Coronaviruses research through an integrated and unified platform.

## Results

### The datasets

#### *Leishmania* parasite datasets

In the present work, we report within the *CidalsDB* platform datasets related to infectious diseases of interest caused by pathogen agents, namely *Leishmania* species and Coronaviruses. For each disease, we performed an extensive search of the literature and retrieved data on molecules with validated anti-pathogen effects. We defined a data dictionary of published information related to the biological activity of the chemical compounds and used it to build the database. Then, we enriched the literature data with confirmatory screening datasets from PubChem. This led to consolidated sets of active and inactive molecules against *Leishmania* parasites and Coronaviruses of high interest in developing ML and DL models for biological activity prediction. Additional infectious diseases will be considered to expand the database content.

**Fig. 1:**
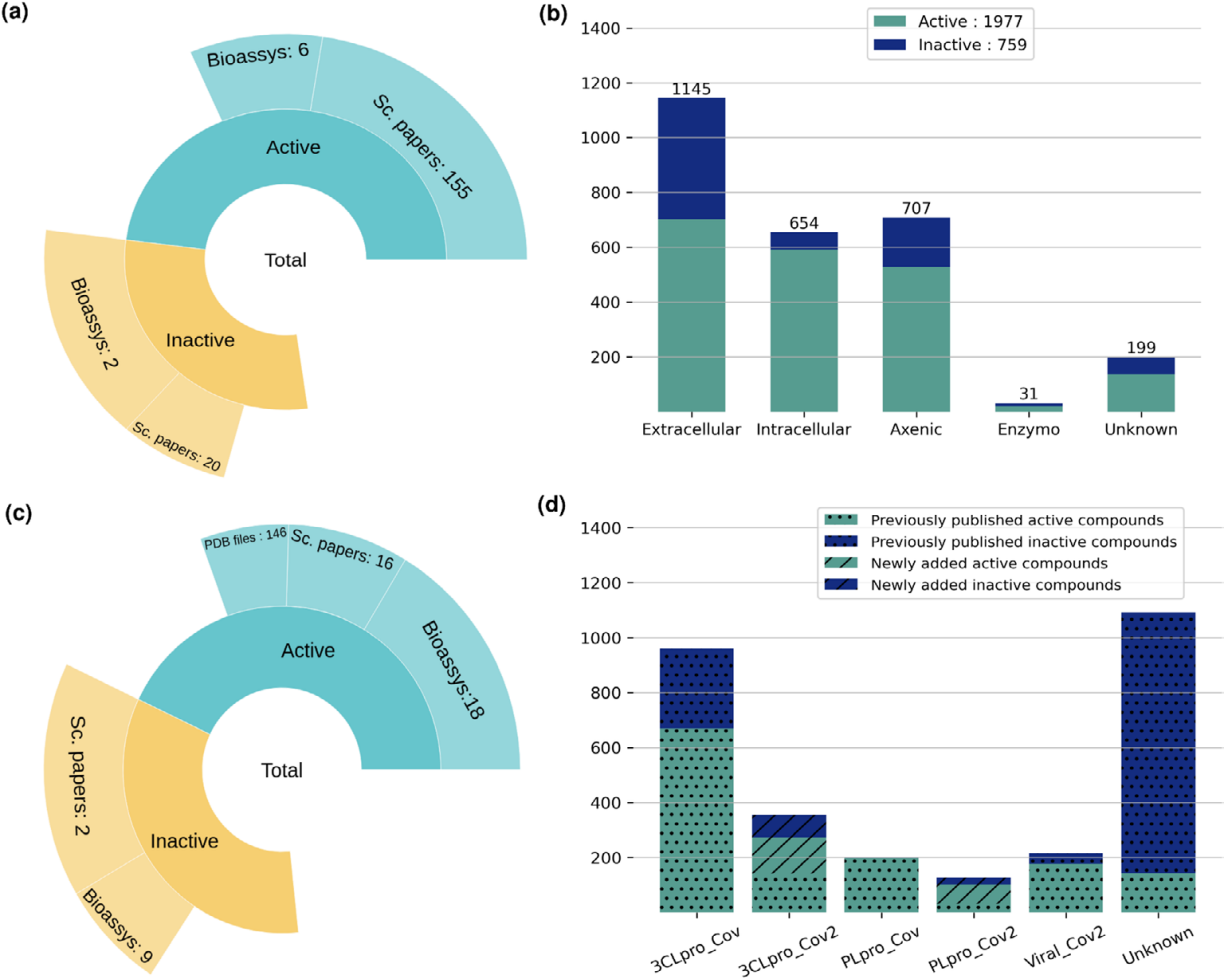
Content of the literature-issued data in *CidalsDB*. **a** Sunburst chart displaying the proportion of anti-*Leishmania* molecules collected from scientific (Sc.) papers and from small biossays to constitute the literature-issued content of *CidalsDB*. **b** Bar plots of the distribution of active and inactive molecules validated at different experimental levels against *Leishmania* parasites. Extracellular denotes the promastigote stage, intracellular denotes the amastigote stage, Axenic denotes the extracellular amastigote-like form of the parasite, Enzymo denotes enzymatic validation stages on known molecular targets. Portions of active molecules are displayed in green and portions of inactive molecules are displayed in blue. **c**) Sunburst chart displaying the proportion of anti-coronaviruses molecules collected from scientific (Sc.) papers, from the PDB database (PDB files) and from small biossays to constitute the literature-issued content of *CidalsDB*. **d** Bar plots of the distribution of active and inactive molecules against Coronaviruses in terms of experimental category, declined into molecules tested against the 3CLpro protease of SARS-Cov (3CLpro_Cov) and SARS-Cov2 (3CLpro_Cov2), molecules tested against the PLpro protease of SARS-Cov (PLpro_Cov) and SARS-Cov2 (PLpro_Cov2), molecules tested on viral growth of SARS-Cov2 (Viral_Cov2), and molecules reported with no specific information on the experimental settings (Unknown). Portions of molecules collected and published in previous work are shown in dotted graphics, and molecules newly collected and added to the anti-coronaviruses dataset are shown in hashed graphics. Portions of active molecules are displayed in green and portions of inactive molecules are displayed in blue

For the *Leishmania* data collection, we fetched 1890 data entries from up to 162 scientific publications, and 846 additional data points from 7 PubChem bioassays. A total number of 2736 data entries divided into 1977 active and 759 inactive molecules were collected (Fig. 1). As some molecules has been described by multiple research papers, the number of unique chemical entities was equal to 2292, out of which 1571 had anti-*Leishmania* effects demonstrated on different species and/or on different stages (in vitro, in cellulo, in vivo) and 721 that had been reported as inactive. This original dataset is first-published in the present research work, and will be referred to as the literature-issued content of *CidalsDB*.

In a second step, we retrieved from the PubChem database, a primary screening bioassay dataset referenced as *AID1063*. It is, to-date the largest dataset reporting anti-*Leishmania* compounds in PubChem, deposited by the University of Pittsburgh Molecular Library Screening Center on February, 29$^{th}$ 2008. It involved 196,173 molecules tested for their inhibitory effects on *Leishmania major* (*L. major*) promastigote growth at 10 $\mu$M concentration in 384-well format assay. Molecules exhibiting > 50% inhibition rate were considered as “Active”. This resulted in 17,630 active and 178,543 inactive molecules, thus an imbalance ratio of 1:9 (Table 1). A second bioassay dataset from PubChem, *AID1258*, was also considered in this study. It is a secondary screening (confirmatory experiments; April, 28$^{th}$ 2008) of a subset of 1,109 molecules out of the *AID1063* bioassay, tested at 1 $\mu$M concentration on *L. major* growth. It resulted in 146 confirmed active molecules (achieving $> 50\%$ inhibition) and 963 confirmed inactive molecules (Table 1).

**Table 1:** Details on the PubChem bioassays used for ML and DL models training: (i) Assay ID number (AID $\hbox {N}^{\circ }$) of the bioassays, (ii) the pathogen targeted through each bioassay, (iii) the size of each bioassay in number of chemical entities tested, (iv) the number of active molecules, (v) the number of inactive molecules, and (vi) the imbalance ratio

AID $\hbox {N}^{\circ }$	Pathogen	Size	Active	Inactive	Imbalance Ratio
AID1063	*Leishmania*	196,173	17,630	178,543	1:9
AID1258	*Leishmania*	1,109	146	963	1:7
AID1706	SARS_COV	291,298	405	290,893	1:717
AID1479145	SARS_COV-2	1,716	73	1,643	1:22

#### Coronaviruses datasets

For the coronaviruses dataset, we considered the previously published data as a starting point, which consisted in 533 molecules from literature consolidated with data from 15 PubChem bioassays, resulting in a total of 2,638 data entries [17]. Molecules included within this dataset were described for their inhibitory effect against one of the three major coronaviruses that caused epidemics, namely the SARS-Cov, MERS and SARS-Cov-2 [17]. Herein, we enriched this dataset with 310 molecules issued from 4 additional PubChem bioassays (Supplementary File S1). These were mainly molecules experimentally validated for their effects against the proteases 3CLpro and PLpro of SARS-Cov2 (Fig. 1.d). As a final figure, we had a curated set of 2,948 entries declined into 1,562 active molecules and 1,386 inactive molecules with a validated effect on at least one pathogen out of the three Coronaviruses (Fig. 1). Only 215 molecules had reported effects against viral growth, 1,314 had inhibitory effects on the main proteases (3CLpro), 327 were effective on the Papain-Like proteases (PLpro) and 1092 molecules were described as anti-coronavirus regardless of the experimental settings (Fig. 1). This updated dataset will be referred to as the literature-issued content of *CidalsDB* for Coronaviruses.

Additionally, we considered two PubChem bioassays *AID1706* and *AID1479145* to be used for the optimization steps of the AI pipeline for the anti-coronavirus molecules identification. The *AID1706* was deposited by The Scripps Research Institute Molecular Screening Center on May, 1$^{st}$ 2009. It is a QFRET-based primary biochemical screening assay to identify inhibitors of the SARS-Cov 3C-like Protease (3CLPro), that resulted in 405 active and 290,893 inactive molecules, thus presenting an imbalance ratio of 1:717. The *AID1479145* is one of the most recent bioassays targeting a SARS-Cov2 protein. It has been deposited by the National Center for Advancing Translational Sciences (NCATS) in June, 22$^{nd}$ 2020. It contains 73 active and 1,643 inactive compounds, tested on the Spike glycoprotein of SARS-Cov2 through a pseudo-typed particle cell entry assay. It presented a lower, yet important, imbalance ratio of 1:22 as compared to the *AID1706* (Table 1).

The collated datasets on both pathogens were heterogeneous as per the collection process. In a previously published research, we demonstrated that heterogeneity did not bias AI models performances in the frame of binary classification of anti-coronavirus molecules [17]. Assuming this hypothesis is true, we herein used data originating from multiple data sources to train and validate ML and DL models for anti-*Leishmania* or anti-Coronaviruses activity prediction.

### Optimal fingerprints embed the least zero values

We sought to optimize the different parts of the ML-based DD pipeline to deliver high performing predictive models. As the encoding system of the chemical information is a central component in the pipeline, we investigated to which extent different fingerprints (FPs) and molecular descriptors, used to generate the input vectors to the ML models, are capturing the chemical diversity of the data. We considered four FPs, namely: RDKit FPs, Atom Pair FPS, Topological Torsion FPs and Morgan FPs; and the molecular descriptors from RDkit to encode the *AID1063* bioassay, as a test case considering its size (196,173 molecules total) and the diversity of its content [23]. We calculated the rate of zeros for multiple vector lengths of the FPs (Table 2 and Supplementary Figure S1). We sought to assess the representativeness of the chemical similarity within the data through the different FPs compared to the molecular descriptors. We estimated this through the performances of a Random Forest (RF) classifier as a baseline model.

The percentage of zeros increased with vector size for all FPs (Table 2). The Morgan FPs and the Topological Torsion FPs presented the highest percentages of zeros, that varied from 72% (256 bits) to 96% (2048 bits) and from 84% (256 bits) to 98% (2048 bits), respectively. The Atom Pair FPs exhibited an average percentage ranging from 43% (256 bits) to 89% (2048 bits). RDKit FPs presented the lowest percentages of zeros overall, that ranged from 5% (256 bits) to 61% (2048 bits).

We then assessed the performances of RF in terms of Precision, Recall and F1-score, when trained on the *AID1063* bioassay encoded by the molecular descriptors and the 4 FPs at the different vector lengths. For all FPs, higher sizes correlated with increased Recall and F1-score (Table 2). The best performances in terms of Recall and F1-score were obtained with the RDKit FPs. The Molecular descriptors led to the lowest Precision overall. Thus, we performed further analysis on the FPs using the 2048 bits length for the upcoming simulations.

**Table 2:** Variation of the FPs composition in zero values with respect to the vector size (in bits) and the subsequent performances of RF in molecule classification based on the different FPs versus the molecular descriptors

Fingerprints	Vector size	% of Zeros	Metrics		
			Precision	Recall	F1-score
RDK	256	5	0.565	0.121	0.200
	512	18	0.622	0.205	0.309
	1024	39	0.650	0.238	0.349
	2048	61	0.643	0.280	0.390
Atom Pair	256	43	0.705	0.0586	0.101
	512	63	0.700	0.080	0.143
	1024	79	0.810	0.114	0.200
	2048	89	0.770	0.128	0.210
Topological Torsion	256	84	0.692	0.128	0.216
	512	92	0.696	0.154	0.252
	1024	96	0.723	0.158	0.259
	2048	98	0.698	0.168	0.271
Morgan	256	72	0.811	0.048	0.091
	512	85	0.807	0.083	0.152
	1024	92	0.758	0.109	0.191
	2048	96	0.766	0.147	0.257
Molecular descriptors	208	–	0.609	0.170	0.274

**Fig. 2:**
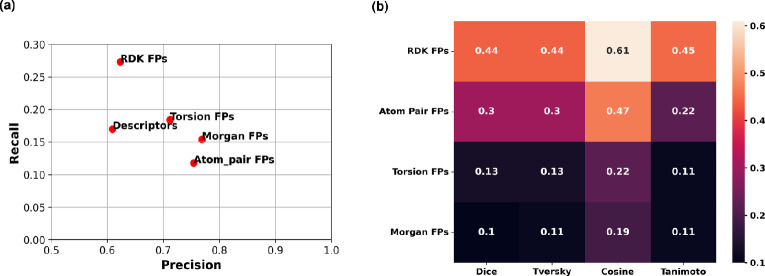
**a** Precision-Recall trade-off of the RF model trained with the different FPs (2048 bits). **b** Chemical similarity assessment of the content of the *AID1063* dataset content through multiple metrics when encoded through the different FPs (2048 bits)

For each 2048-bit FP and the molecular descriptors, we assessed the Precision-Recall trade-off of the RF model. The Morgan FPs and Atom Pair FPs presented the highest Precision, with low Recall values. The Topological Torsion FPs had the least interesting Precision-Recall trade-off, while the RDKit FPs exhibited an optimal Precision-Recall trade-off (Fig. 2a).

To better assess the effectiveness of each FP in reflecting and capturing the chemical diversity within the dataset, we calculated the pairwise distance between all molecules within the *AID1063* dataset encoded by each FPs. Four distance metrics were used, namely Sørensen-Dice, Tversky, Cosine and Tanimoto coefficients. The average values for all metrics were the lowest for the Morgan and Topological Torsion FPs, followed by the Atom Pair FPs (Fig. 2b). RDKit FPs, on the other hand, led to higher average distance values through all metrics. Taking into account the significantly low percentage of zeros within the RDKit FPs compared to the other FPs for a given vector length (Table 2, Supplementary Figure S1), we concluded that the RDKit FPs optimally captured the chemical diversity within the *AID1063* dataset, and were used throughout the subsequent analysis.

### Enrichment through *CidalsDB* data outperforms state-of-the-art balancing methods

We focused on four machine learning (ML) and three deep learning (DL) algorithms of interest, namely: Random Forest (RF), Multi-Layer Perceptron (MLP), Gradient Boosting (GB), Naive Bayes (NB), Graph-Convolution Network (GCN), Message-Passing Neural Network (MPNN) and ChemBERTa. We sought to identify the algorithms that can optimally predict the “cidal” effect of chemical molecules against *Leishmania* parasites and Coronaviruses. We trained the models on datasets from highly imbalanced PubChem bioassays, as follows: *AID1063* and *AID1258* for *Leishmania* parasites and *AID1706* and *AID1479145* for coronaviruses. These datasets present high imbalance ratios towards the inactive class. Thus, we assessed the impact of data balancing through augmentation or reduction of data points on the different algorithms’ performance in a second step. Last, we explored how enriching these datasets with the literature-issued content of *CidalsBD*, as a non-synthetic data balancing technique, could enhance the activity prediction outcome of the models.

**Fig. 3:**
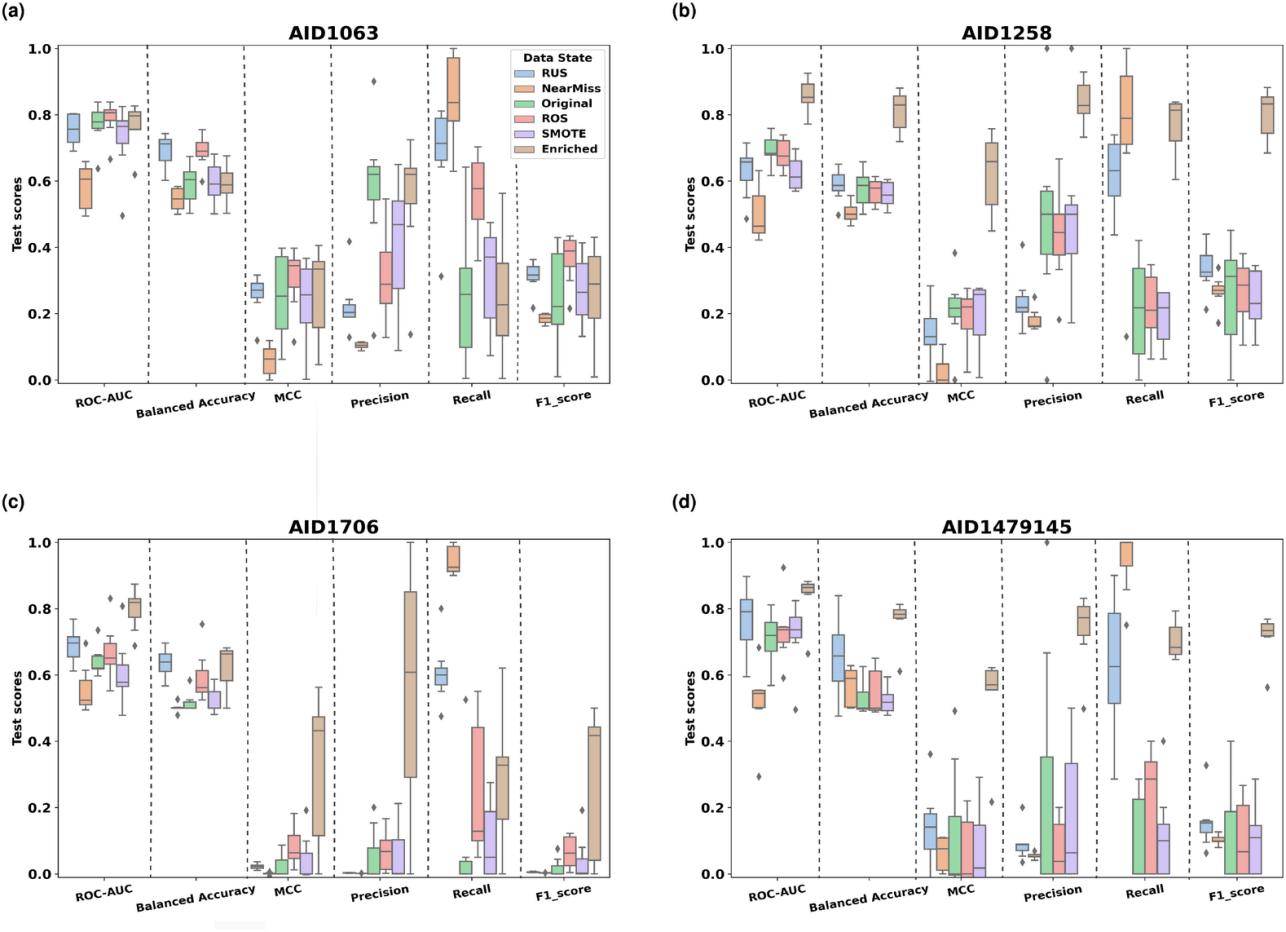
Algorithms’ performances achieved by the different models (4 ML and 3 DL), when trained on the 6 versions of each bioassay (Original, RUS, NearMiss, ROS, SMOTE and Enriched). **a** Boxplot of all models’ performances according to the considered version of the *AID1063* dataset. **b** Boxplot of all models’ performances according to the considered version of the *AID1258* dataset. **c** Boxplot of all models’ performances according to the considered version of the *AID1706* dataset. **d** Boxplot of all models’ performances according to the considered version of the *AID1479145* dataset

For the leishmaniasis data, training all algorithms on the highly imbalanced dataset from PubChem bioassay *AID1063* (1:9 imbalance ratio) led to the highest Precision values as compared to balanced versions of the same dataset through synthetic resampling (Fig. 3a). The random oversampling technique (ROS) enhanced the Recall significantly and the Balanced Accuracy moderately. It decreased the Precision as a drawback, but did not affect the ROC-AUC and the MCC scores as compared to the original dataset. The SMOTE oversampling performed poorly as compared to ROS on this dataset, with Recall, MCC and Balanced accuracy comparable to the original imbalanced dataset (Supplementary File S2). The random undersampling (RUS) method significantly enhanced the Recall, with a significant decrease of the Precision. RUS led to the highest Balanced Accuracy overall, with a slight decrease of ROC-AUC and MCC scores. The NearMiss technique achieved the highest Recall values and the lowest values of all remaining metrics. This indicated that the synthetic resampling methods did not enhance the classification power for this dataset. Comparable metric values and performance trends were observed with the *AID1258* bioassay (1:7 imbalance ratio) (Fig. 3b).

In a second step, we assessed model performances on the same bioassays (*AID1063* and *AID1258*) enriched with the manually collected anti-*Leishmania* dataset of *CidalsDB*. The enrichment did not enhance the models performances for the *AID1063* dataset (Fig. 3a). This is an anticipated result due to the low percentage of added data points to the original dataset, considering its size. In fact, the enriched *AID1063* presented the same imbalance ratio as the original dataset (1:9). On the other hand, enrichment of the *AID1258* led to enhancement of all metrics (Fig. 3b). Interestingly, both the Recall and the Precision were significantly increased, and the trade-off previously observed in all other cases could be subdue.

For the coronavirus dataset, as we compare the algorithms’ performances when trained on the original bioassay datasets versus the undersampled and the oversampled versions of the data, we can only report a slight increase of the Balanced Accuracy and the ROC-AUC, and a significant enhancement of the Recall for both bioassays *AID1706* and *AID1479145* (Fig. 3c, d). Overall, we observed lower performances as compared to the *Leishmania* datasets, due to higher imbalance ratios of 1:717 and 1:22, respectively. This could explain the overall low performances. Interestingly, for the coronavirus datasets, enrichment through *CidalsDB* content led to significant enhancement of models’ performances. This is significantly more marked for the *AID1479145* dataset (Fig. 3d).

Overall, oversampling, which consists as a general figure in randomly or synthetically duplicating “active” molecules to the dataset, induced minimal enhancement of models’ performances, with the critical drawback of reducing the Precision (i.e. reduced power of correctly predicting the “active” class). Also, undersampling, which consists in omitting data from the “inactive” class and inducing information loss, reduced the models’ performances overall (Fig. 3). The enrichment specifically proved effective on the smallest datasets *AID1258* and *AID1479145*, as it addressed the imbalance issue through non-synthetic data augmentation and the embedding of informative data points within the “active” class towards enhanced predictive power (Supplementary File S3).

### Pathogen-specific combinations of AI models and enriched datasets exhibited optimal predictive performances

#### Best {model-dataset} combinations

**Fig. 4:**
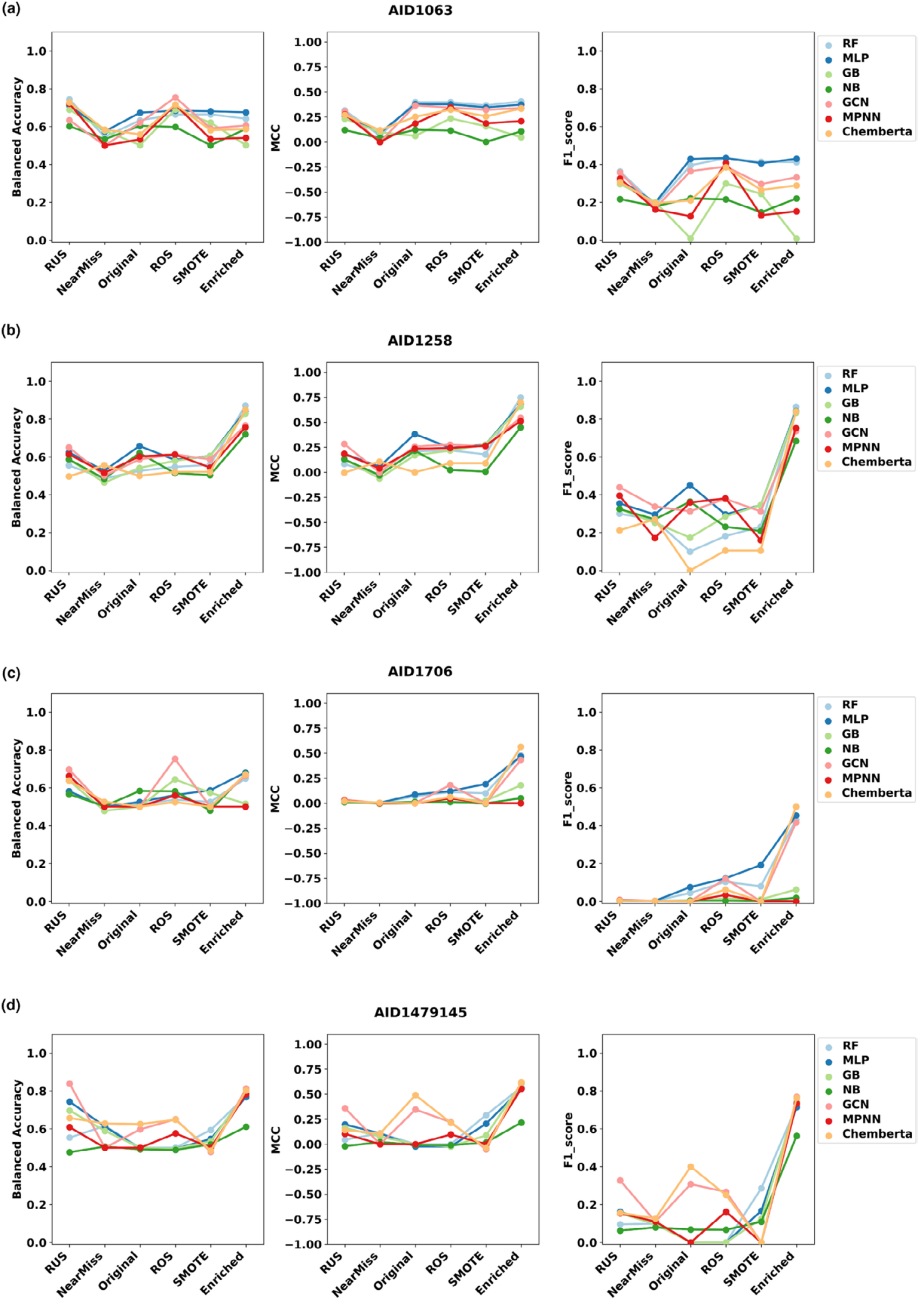
Effects of the different synthetic resampling methods and the enrichment approach on the ML and DL algorithms’ performances based on three metrics: Balanced Accuracy, MCC and F1-score. **a** Models performances when trained on the different variations of the *AID1063* dataset. **b** Models performances when trained on the different variations of the*AID1258* dataset. **c** Models performances when trained on the different variations of the*AID1706* dataset. **d** Models performances when trained on the different variations of the*AID1479145* dataset

Comparison of the models’ performances across the different settings of the datasets, i.e. undersampled (RUS, NearMiss), original, enriched, oversampled (ROS, SMOTE) revealed a significant performance increase of all models trained on the enriched datasets (Fig. 4). In fact, the enrichment led to better results as compared to the imbalanced dataset and both oversampled dataset (ROS and SMOTE). Interestingly, it addressed the Precision-Recall trade-off otherwise observed. In fact, all models achieved their highest scores in Precision and Recall when trained on the enriched datasets. This is translated by a peak on the corresponding F1-score panel on Fig. 4, except for the *AID1063* (Fig. 4.a). We particularly focused on the enriched versions of the *AID1258* and *AID1479145*, as these datasets inferred optimal performances of the ML and DL models (Supplementary Figures S2 & S3).

With *AID1258*, enrichment derived significant enhancements of the MCC, Precision, Recall and F1-score (Table 3). For RF and GB models, Recall and F1-score achieved increments ranging from +0.48 to +0.76. Performances of MLP were also significantly increased through enrichment to reach 0.86 and 0.83 for Precision and Recall, respectively. GCN and MPNN presented moderate enhancement of their performances, and NB remained the least performing model on this dataset. Interestingly, ChemBERTa demonstrated the highest enhancement of performances that reached + 0.93, + 0.84 and + 0.88 for Precision, Recall and F1-score respectively. In fact, this model exhibited zero values for MCC, Precision, Recall and F1-score when trained on the original *AID1258* dataset. Overall, RF and ChemBERTa achieved the highest performances with exceptional MMC values of 0.74 and 0.76, highlighting the power of these models to accurately distinguish the active from the inactive molecules. This was backed up by the highest F1-scores reaching 0.86 and 0.88, respectively (Fig. 4.b).

With *AID1479145*, GCN and ChemBERTa appeared to be the top performing algorithms (Fig. 4d). They both achieved MCC scores of 0.62. They also exhibited comparable Recall values of 0.79 and 0.71, Precision values of 0.74 and 0.83, leading to the highest F1-scores of 0.77 and 0.76, respectively (Table 3). Alongside GCN and ChemBERTa, the GB model also achieved interesting performances with an MCC score of 0.60, a Recall of 0.68, a Precision of 0.82 and a F1-score of 0.74. MPNN demonstrated similar performances with inverted values of the Recall and the Precision and a least interesting MCC score (0.55) as compared to the GB model. RF and MLP exhibited lower performances as compared to GCN and GB, and to their performances on the *Leishmania* dataset. The NB model achieved the least interesting performances overall (Table 3).

To conclude, RF, MLP and ChemBERTa appeared to be the best predictive models when trained on the enriched version of the Leishmanaisis dataset, while GCN, GB and ChemBERTa were identified as the best models when trained on the enriched version of the Coronaviruses dataset. Thus, we proceeded to optimize the performances of these pathogen-specific models towards their deployment on *CidalsDB*, the web application, alongside the corresponding enriched datasets.

**Table 3:** Impact of the enrichment of the *Leishmania* (AID1258) and the *Coronaviruses*(AID1479145) bioassays with *CidalsDB* content on the performances of the ML and DL algorithms in molecule classification

Dataset	Model	Balanced accuracy	ROC-AUC	MCC	Precision	Recall	F1-score
AID1258 Enriched with Cidals	RF	**0.87** (*+ 0.34*)	**0.90** (+ 0.22)	**0.74** (*+ 0.53*)	**0.92** (− 0.07)	**0.81** (*+ 0.76*)	**0.86** (*+ 0.76*)
	MLP	**0.84** (+ 0.18)	**0.85** (+ 0.11)	**0.68** (*+ 0.30*)	**0.86** (+ 0.27)	**0.83** (*+ 0.46*)	**0.84** (*+ 0.39*)
	NB	**0.71** (+ 0.1)	**0.77** (+ 0.09)	0.44 (+ 0.23)	**0.78** (*+ 0.46*)	0.60 (+ 0.18)	0.68 (*+ 0.32*)
	GB	**0.82** (+ 0.28)	**0.88** (+ 0.26)	**0.65** (*+ 0.48*)	**0.82** (*+ 0.32*)	**0.83** (*+ 0.73*)	**0.83** (*+ 0.66*)
	GCN	**0.76** (+ 0.18)	**0.85** (+ 0.16)	0.54 (+ 0.28)	**0.82** (+ 0.26)	0.67 (*+ 0.45*)	**0.73** (*+ 0.42*)
	MPNN	**0.76** (+ 0.16)	**0.82** (+ 0.11)	0.51 (*+ 0.32*)	**0.73** (*+ 0.30*)	**0.77** (*+ 0.40*)	**0.75** (*+ 0.39*)
	ChemBERTa	**0.88** (*+ 0.38*)	**0.93** (*+ 0.36*)	**0.76** (*+ 0.76*)	**0.93** (*+ 0.93*)	**0.84** (*+ 0.84*)	**0.88** (*+ 0.88*)
AID1479145 Enriched with Cidals	RF	**0.77** (+ 0.14)	**0.85** (+ 0.27)	0.57 (*+ 0.57*)	**0.79** (*+ 0.79*)	0.66 (*+ 0.66*)	**0.72** (*+ 0.72*)
	MLP	**0.77** (+ 0.27)	**0.84** (+ 0.06)	0.55 (*+ 0.57*)	**0.77** (*+ 0.77*)	0.66 (*+ 0.66*)	**0.71** (*+ 0.71*)
	NB	0.61 (+ 0.12)	0.66 (+ 0.09)	0.22 (+ 0.22)	0.50 (*+ 0.46*)	0.64 (*+ 0.36*)	0.56 (*+ 0.49*)
	GB	**0.79** (+ 0.29)	**0.87** (+ 0.13)	**0.60** (*+ 0.60*)	**0.82** (*+ 0.82*)	0.68 (*+ 0.68*)	**0.74** (*+ 0.74*)
	GCN	**0.81** (+ 0.21)	**0.88** (+ 0.16)	**0.62** (+ 0.27)	**0.74** (+ 0.08)	**0.79** (*+ 0.60*)	**0.77** (*+ 0.46*)
	MPNN	**0.78** (+ 0.28)	**0.86** (+ 0.23)	0.55 (*+ 0.55*)	0.69 (*+ 0.69*)	**0.78** (*+ 0.78*)	**0.73** (*+ 0.73*)
	ChemBERTa	**0.80** (+ 0.18)	**0.87** (+ 0.06)	**0.62** (+ 0.12)	**0.83** (− 0.17)	**0.71** (*+ 0.46*)	**0.76** (+ 0.26)

Values of balanced accuracy, ROC-AUC, MCC, Precision, Recall and F1-score achieved by the different models (RF, MLP, NB, GB, GCN, MPNN and ChemBERTa) when trained on the enriched datasets are shown in the corresponding boxes. In cases where the performances were enhanced due to the enrichment, the increase rate is shown as a positive shift between brackets. Positive shifts higher than 0.3 are shown in italics. In cases where the performances were decreased due to the enrichment, the decrease rate is shown as a negative shift between brackets (in underline). Metrics values higher than 0.7 are shown in bold, except for MCC where the threshold is 0.6

#### Models optimization

In order to enhance the robustness of our models, we performed a grid search to identify the optimal hyper-parameters of the best performing models trained on the enriched *AID1258* and *AID1479145* datasets. The models were evaluated based on the hyper-parameter combinations that maximized the Balanced accuracy and the F1-score (Supplementary File S4). Furthermore, we performed a 5-fold cross-validation of all models’ performances that demonstrated consistent performances (Table 4).

We also included in Table 4 performances of the RF and the GCN models from previous publications by our group [17, 23]. The RF was previously trained on a reduced version of the *AID1063* and successfully identified novel anti-*Leishmania* drug candidates [23, 24]. Due to the imbalance state of the bioassay, it only achieved a precision of 0.23 and a F1-score of 0.37. The Recall and balanced accuracy were 0.84 and 0.72, respectively [23]. In the present work, RF achieved a Recall of 0.3, a Precision of 0.65, a F1-score of 0.41 and a balanced accuracy of 0.64 when trained on the enriched version of the *AID1063* (Supplementary File S2). Through the enrichment of the *AID1258*, RF could achieve higher performances with allmetrics $> 0.80$, which supports the relevance of such a strategy towards enhanced AI-based biological activity predictions of chemical compounds (Table 3). Similarly, we included the performances of the GCN model, previously trained on a reduced version of the literature-issued content of CidalsDB on Coronaviruses [17]. The enrichment strategy permitted to moderately enhance the Recall (+ 0.07) and F1-score (+ 0.04) of the model, with no significant fluctuation of the precision ($-$0.01).

The enrichment strategy outperformed all synthetic resampling methods. Nonetheless, it proved to be efficient for the *AID1258*, *AID1479145* and *AID1706*; and presented limited effect on the *AID1063* (Fig. 4). For the small datasets *AID1258* and *AID1479145*, the enrichment achieved a high balancing effect, as they reached imbalance ratios close to 1:1. For the large bioassays *AID1063* and *AID1706*, the enrichment did not enhance the balance ratio due to their initial sizes. Yet, performances were significantly enhanced for some models with the *AID1706*, but not with the *AID1063*. With regards to these results, we could not deduce that the imbalance ratio is the major limiting characteristics towards high performances of models trained on these large datasets. The content of each bioassay and the diversity of the data entries shall play a pivotal role in the prediction outcomes.

**Table 4:** Models’ performances through a 5-fold cross-validation

Model	Balanced Accuracy	Precision	Recall	F1-score
RF_*Leishmania*	0.84 ± 0.01	0.90 ± 0.02	0.75 ± 0.01	0.82 ± 0.02
RF_*Leishmania*(2022 [23])*	0.72	0.23	0.84	0.37
MLP_*Leishmania*	0.81 ± 0.03	0.80 ± 0.05	0.83 ± 0.03	0.82 ± 0.02
ChemBERTa_*Leishmania*	0.84 ± 0.02	0.91 ± 0.05	0.77 ± 0.05	0.82 ± 0.02
GCN_Coronavirus	0.81 ± 0.01	0.76 ± 0.03	0.77 ± 0.04	0.77 ± 0.01
GCN_Coronavirus (2021 [17])	N/A**	0.77 ± 0.06	0.70 ± 0.08	0.73 ± 0.04
GB_Coronavirus	0.80 ± 0.01	0.79 ± 0.02	0.71 ± 0.03	0.75 ± 0.01
ChemBERTa_Coronavirus	0.82 ± 0.01	0.80 ± 0.03	0.76 ± 0.04	0.77 ± 0.01

Performances of the RF, MLP and ChemBERTa models trained on the enriched bioassay *AID1258* are reported alongside the previously published performances of the RF model trained on an anterior version of the *AID1063* [23]. Performances of the GCN, GB and ChemBERTa models trained on the enriched bioassay *AID1479145* are reported alongside the previously published performances of the GCN model trained on an anterior version of the coronaviruses-related literature-issued content of *CidalsDB* [17]

$^{*}$ RF performances were published in 2022 without the standard deviation values

$$^{**}$$ GCN performances were published in 2021 with no balanced accuracy reported.

N.B: The dataset was balanced and authors computed the model’s accuracy = 0.79 through a 10-fold cross-validation

### The platform *CidalsDB*

#### *CidalsDB*: The database

The *CidalsDB* relational database consists of six tables: Compound, Pathogen, Target, Test_type, Tested and Enzymo. Detailed description of the content is provided in (Supplementary Figure S4). The Compound table includes all data intrinsically related to the compounds. These data belong to five categories: identifiers, chemical structure, physicochemical properties, activity-related information, and pharmacokinetic and pharmacodynamic properties. The Pathogen table includes data on the pathogen organism namely: taxid, species, strain, clinical form, and caused disease(s). The Target table includes data on the molecular targets when known, which are mainly proteins. It includes the UniProt ID, the PDB ID if any, size (length in amino acids), the protein class, and the essentiality if demonstrated. The Test_type table contains the type of experimental tests that have been performed to assess the compound effects. These can be: extracellular, intracellular, axenic, host_cell, in_cellulo and in_vivo. The Tested table was conceived as an association between tables Compound, Pathogen and Test_type. Each compound could have been tested on a given pathogen in different types of experiments, i.e on its extracellular or intracellular form, on the host cell, in vivo, or in any other experimental settings. The Enzymo table contains data relative to the action of a compound on a given protein target. It contains a Uniprot identifier of the target and the enzymatic and/or biochemical experimental data of the compound activity, among other information.

**Fig. 5:**
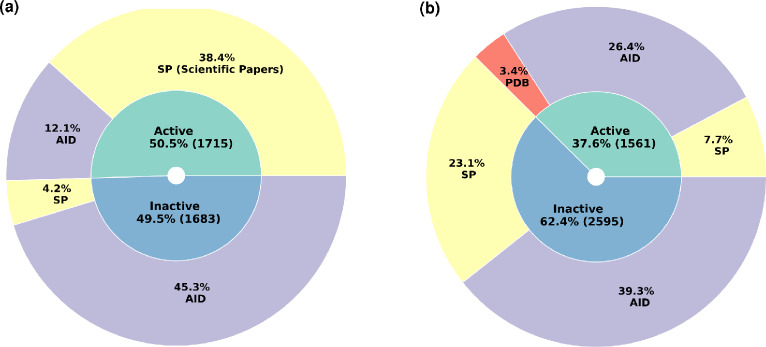
Content of *CidalsDB*. **a** Statistics of the deployed versions of the enriched *AID1258* bioassay. **b** Statistics of the deployed versions of the enriched *AID1479144 * bioassay

Datasets described in the present work as leading the optimal performances of the ML and DL algorithms were made accessible for browsing through the web interface *CidalsDB*. These corresponded to the enriched versions of the bioassays *AID1258* and *AID1479145*. As a final figure, the *Leishmania* dataset enclosed 3845 entries corresponding to 3398 unique molecules, out of which 1715 (50.5%) are active and 1683 (49.5%) are inactive (Fig. 5a). The Coronaviruses dataset contained 4220 entries corresponding to 4156 unique molecules divided into 1561 (37.6%) active and 2595 (62.4%) inactive compounds (Fig. 5b). Thus, the deployed dataset encompasses 8,065 chemical entities including both pathogen groups.

#### *CidalsDB*: The web interface

The web interface of the platform *CidalsDB* was conceived to be as simple as possible. An explicative video was prepared as a walkthrough tutorial (See tutorial here). The interface included five tabs as follows:

***HOME*** The HOME tab offers multiple information about the *CidalsDB* platform. Updated status of the database content is displayed through dynamic bar plots and pie charts. Based on the optimal results obtained through the enrichment strategy, we retained the enriched *AID1258* and *AID1479145* as final figures of *CidalsDB* content (Fig. 5).

**Fig. 6:**
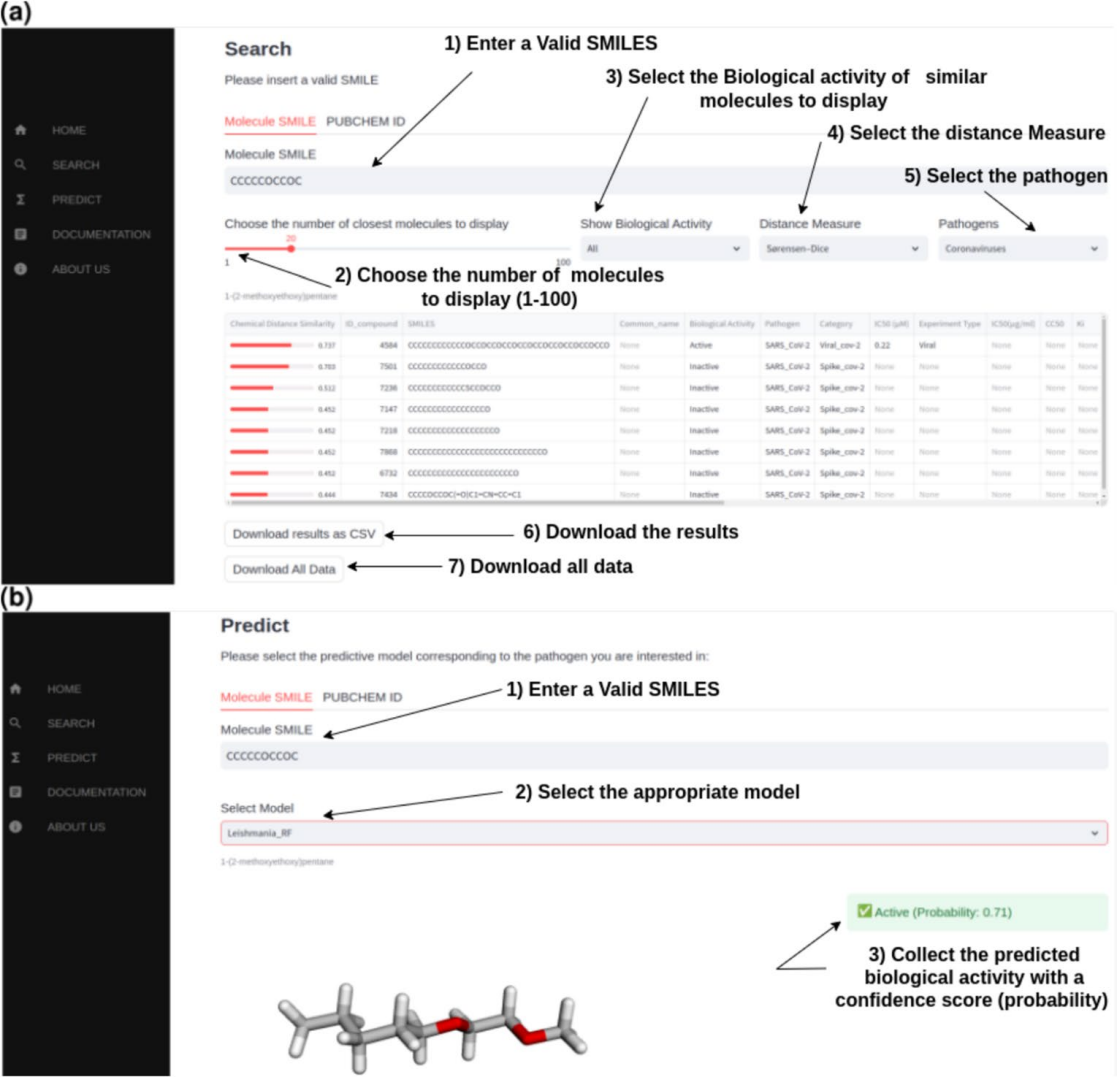
The *CidalsDB* interface. **A** A screenshot of the SEARCH tab of the web interface displaying a search results to a SMILE query. **B** A screenshot of the PREDICT tab of the web interface returning a positive prediction (“Active”) for the query molecule using the *Leishmania_RF* model

***SEARCH*** The SEARCH tab on the web interface allows for database browsing. The user may enter a PubChem ID, a SMILES or a CSV file containing multiple SMILES entries to search the database for molecules chemically similar to the query compound(s). This permits an easy browsing of the database using one query molecule or batch processing of multiple molecules. Browsing can be performed against the *Leishmania* dataset, the coronaviruses dataset or both. Up to 100 compounds from *CidalsDB* can be displayed in a tabular form, sorted in descending order based on their chemical similarity values assessed through a chosen metric out of the Tanimoto coefficient, the Sørensen-Dice coefficient, the Cosine coefficient, the Tversky coefficient, or the average of all these metrics (Fig. 6a). The search results can be downloaded as a CSV file, containing the molecules ID, their chemical structure encoded as SMILES and activity data against a specific disease, alongside all available information on that molecule in the database. Additional information include but are not limited to: origin of the compound, chemical formula, molecular weight, experimental data at different stages (in vitro, in vivo), etc.

***PREDICT*** The PREDICT tab on the web interface gives access to a no-code AI-based prediction of the query molecule(s) activity against *Leishmania* parasites using the RF, the MLP or the ChemBERTa models, and against Coronaviruses using the GCN, the GB or the ChemBERTa models (Fig. 6b). The molecule corresponding to the SMILES entry will be assessed for its anti-*Leishmania* or anti-coronaviruses potential through any model within the dropdonw list. The prediction results will be instantly obtained as a classification into “Active” or “Inactive” with a confidence score ranging from 0 to 1. The confidence score is the probability returned by the predictive model of the molecule being effectively active or inactive.

***DOCUMENTATION*** The DOCUMENTATION tab encloses all necessary information about the metrics and the encoding system of the chemical entities used through the SEARCH functionality. It also provides details on the ML and DL models deployed as part of the prediction functionality and accessible within the PREDICT tab.

***ABOUT US*** The ABOUT US tab offers information about the scientific team behind the *CidalsDB* platform, as well as a simple form to send messages in case of specific inquiries.

The *CidalsDB* platform was conceived as a scalable system for chemical data browsing and fetching in the frame of anti-pathogen CADD research. Its AI-based prediction tools were developed, fine-tuned and optimized for each pathogen separately, taking into consideration the specificity of the experimental data for each pathogen model, data availability and scalability.

## Discussion

With the rise of AI technologies and the growing access to large bioassay datasets, the AI-assisted drug discovery field witnessed an unprecedented expansion. Research efforts concerned collecting, homogenizing and storing data into databases, developing novel AI algorithms and methods, deploying software and web server for democratized access to data and AI technologies, among others. The present work is a contribution to the development and democratization of AI-assisted drug discovery against infectious diseases. Our research delivered multiple outcomes that are essential to advancing drug discovery, namely: (i) Open access curated datasets against *Leishmania* parasites and coronaviruses; (ii) A web-based interface for database content browsing using chemical similarity and (iii) Six pre-trained ML and DL models optimized for predicting biological activity against the pathogens of interest.

The datasets in *CidalsDB* were collated from various sources and manually curated with the primary objective of deriving high performing AI models for biological activity prediction. The web interface was conceived as a user-friendly platform, through which non-computational scientists can seamlessly perform activity prediction of one or multiple query molecules using high performing pre-trained ML and DL models.

A few platforms focusing on AI-assisted prediction of anti-pathogen activity of molecules of interest to non-computational scientists could be identified. We herein cite DeepScreening, a web-based platform that encompasses a customizable MLP model construction feature and permits model training on a user-provided dataset or on ChEMBL bioassays to predict interactions between drug candidates and biological targets [25]. Similarly to *CidalsDB*, DeepScreening enables scientists to access no-code ML models. Nonetheless, users of DeepScreening should get familiar with basic mathematical notions to be able to implement the model (parameters choice). On the other hand, the DeepScreening server is not restricted to specific pathogens or pathologies like *CidalsDB*, making it of broader interest. The user can upload a dataset with a specific focus for the model training as a SDF file containing a maximum of 3000 molecules. Basic skills and knowledge on chemical data formatting are required in order to constitute a valid input file of the dataset.

### From a database perspective

The web interface of *CidalsDB* enables browsing of datasets of chemical entities with validated biological activity against the pathogens of interest. The chemical similarity searching functionality presented the advantage of browsing for molecules with no known IDs in reference databases and/or molecules newly identified or generated by the user(s). The multiple similarity metrics were implemented to enlarge the coverage range of the search. The users can directly examine the search results on the web interface, or download them as a comma-separated file (CSV format).

Multiple research groups have contributed to the field through specialized databases and datasets [11, 13, 15, 26]. The *LeishInDB* is a database of 8,273 anti-*Leishmania* compounds that can be searched by filtering on biological activity types and parasite strains [13]. It presents limitation of access to data as users can only download the search results within the limit of 1000 molecules. Other specialized databases providing datasets for the DD research require registration and limitations of access [26, 27]. Nonetheless, there are incremental efforts to develop open access datasets dedicated to DD research with a focus on data collection and curation. SMACC (Small Molecule Antiviral Compound Collection) is a very interesting initiative deploying a collection of 32,500 entries for 13 viral infections using ChEMBL as the main data source [28]. The datasets are open source and available for direct download as excel sheets, with no web-interface for data browsing online. Such a resource is valuable for extending the *CidalsDB* platform, in future work, through enriching the Coronaviruses dataset or through including additional viral infections of interest. The web interface of *CidalsDB* would then offer the data browsing functionality of such datasets.

### From AI models’ perspective

In addition to the datasets as first outcomes of *CidalsDB*, the present work contributes with pre-trained AI models validated for biological activity prediction tasks against two groups of infectious pathogens, with the perspective to cover additional diseases. Multiple research efforts have led to the prediction and validation of novel anti-pathogen compounds using machine learning or deep learning approaches [17, 23, 24, 29–31]. Classically, predictions of biological activity and other properties of interest, called Structure-Activity Relationship (SAR) modeling, are performed through statistical models. As an example, we cite the PASS Online, a web server that performs predictions based on SAR data for more than 18,977 substances described for at least one of 124 biological activity, including anti-protozoan (*Leishmania*) and anti-viral (coronaviruses) [32]. The approach was rooted on the Multilevel Neighborhoods of Atoms (MNA) descriptors [33], that accurately quantify molecular features and enables the subsequent statistical model to predict whether a compound might exhibit a given biological activity. The web server can be used for free, but registration is mandatory. The PASS Online platform provides for a given query molecules the different possible biological activities it may have, within the categories covered by the training set [32, 34]. It presents the advantage of providing all possible biological activities of a chemical entity with a confidence score. Through *CidalsDB*, we also included the confidence score with the prediction outcome for a query molecule. This feature is highly valuable for explainable and interpretable results from the user’s perspective.

More recently, a few platforms deploying ML and DL models trained for specific tasks have been published. Most of these focused on ADMET (Absorption, Distribution, Metabolism, Excretion, and Toxicity) predictions, namely ADMET-AI [35], DeepPK [36], SwissADME [37], ADMET-boost [38] and ADMET-lab 2.0 [39]. The DeepPK platform used a Directed Message Passing Neural Network (D-MPNN), a variation of the MPNN model [40], for ADMET prediction. It achieved a ROC-AUC score of 0.75 when trained on a dataset of 456,331 entries issued from ChEMBL bioassays [36]. We herein reported ROC-AUC scores varying from 0.61 to 0.75 for the MPNN model trained on the original imbalanced datasets. These performances reached 0.86 when trained on the enriched versions of the bioassays. Noticeably, it achieved comparable performances to the GB on the coronaviruses dataset, but couldn’t be identified as a top performing model in the present work due its moderate MCC (0.55) and Precision (0.69) scores (Table 3, Fig. 4 and Supplementary Figure S2). This highlighted the importance of tailoring one model performances on a particular, yet of high quality dataset, and for a specific task.

One key result from the present work is the use of data enrichment as a balancing/resampling technique. This strategy represents a novel approach in the field, addressing limitations commonly encountered with highly imbalanced datsets in drug discovery. While traditional methods such as ROS, RUS, NearMiss, SMOTE, ADASYN [41] among others are frequently employed to handle imbalance issues [42], we demonstrated that data enrichment with literature-issued entries significantly enhanced model performances. Noticeably, we could not confirm that higher imbalance ratios strictly correlate with poor performances. The enrichment of the *AID1063* bioassay, although presenting a relatively low imbalance ratio (1:9) as compared to the *AID1706* bioassay (1:717), did not lead performance enhancement (Fig. 4). Intrinsic characteristics of the dataset should be further explored towards understanding the underlying results.

The *CidalsDB* platform presents the special feature of deploying three pre-trained and optimized models for each group of pathogen. Through several simulations that involved a multitude of conditions on four datasets in different states (original, resampled, enriched), we trained seven ML and DL models. There were no direct evidence on the supremacy of ML models over DL models or *vice versa*. Nevertheless, our results confirmed that data wrangling and balancing were key elements to achieve satisfactory performances of the trained models.

## Conclusion

To conclude, our work consisted in deploying *CidalsDB* a novel ready-to-use resource for the development of AI-assisted DD against *Leishmania* parasites and Coronaviruses. To our knowledge, this is the first resource that offers the browsing, access and download of the data alongside the functionality of no-code use of pre-trained AI models for DD purposes. *CidalsDB* is made publicly available through the web and it was conceived towards an expansive platform to include additional pathogens of interest.

## Methods

### Database design and implementation

Through a primary screen of the literature, we defined a set of data that occurred in research articles dealing with the anti-pathogenic effect of chemical compounds. This permitted the definition of a data dictionary. Data was then structured into tables of data and relations according to a conceptual data model (CDM). The CDM was then checked for discrepancies and translated into a logical data model (LDM). We generated the SQL code to implement the generated LMD. The SQL code served to implement the relational database under the software tool PhpMyAdmin [43].

### Data fetching and entry

For each pathogen of interest, data was collected manually through an extensive search of the literature using the pathogen name and the disease it causes associated with multiple combinations of the following keywords: metabolite/compound, activity, natural/synthetic, taxonomy, target, therapeutic, drug among others. Data from related databases including Uniprot [2], the RCSB PDB [8], the Zinc database [3], ChemSpider database [4], ChEBI database [5] and PubChem [6, 7] were also fetched and complemented as needed from the literature. This primary data was entered and managed using the PhpMyAdmin graphical interface [43].

In a second step, for each pathogen of interest, subsets from bioassays, deposited in the PubChem database, resulting from high throughput screenings were integrated, to complement the database content. The compound identifier (CID), the SMILES entry coding the chemical structure, at least one column on activity results (IC50, EC50, Inhibition % at a given concentration) and the activity outcome (active, inactive, unspecified or inconclusive).

### Data standardization

Molecules collected from the literature with a reported anti-pathogen activity (IC50, EC50, IC90, etc) were labeled as “Active” and those reported in the literature as non active were labeled as “Inactive”. The integrity of the SMILES of literature-issued molecules was verified, and broken SMILES were corrected. Molecules issued from the PubChem bioassays detained a class label as part of the available information. Only molecules labeled as “Active” or “Inactive” were retained as part of the input datasets for the AI models training. Integration of data from both sources were accomplished and duplicate removal was performed.

### Web interface development

We customized the Streamlit library in order to implement a user-friendly web interface that integrates key functionalities related to the datasets and the molecules activity prediction. The Streamlit library makes it easy to implement various Python libraries of interest to our models and data analysis approaches, such as Scikit-learn, DeepChem, Pandas, RDKit, and Plotly.

### Data encoding

Data encoding was performed using the RDKit [44] and the DeepChem [45] libraries under Python. We used molecular fingerprints (FPs) to generate input vectors to the machine learning (ML) algorithms. We used graph convolution approaches using the *MolGraphConvFeaturizer* method under DeepChem to generate input vectors to the graph-based deep learning (DL) models. Finally, we used the *Tokenizer* method developed by the Hugging Face [46] to generate the input vectors for the transformer-based model.

For the selection of the best encoding system for the ML models, we tested the impact of four FPs, namely the RDKit FPs, the Atom Pair FPs, the Topological Torsion FPs and the Morgan FPs with radius 2 (ECFP4). For each FP, four vector sizes were generated: 256, 512, 1024 and 2048 bits. We then trained and evaluated the performances of the Random Forests (FR) classifier on the *AID1063* bioassay, using a 80/20 train-test split to assess the impact of the vector size on the model performances. We reported values of the Precision, Recall and F1-score values for each simulation.

### Machine learning and deep learning algorithms

We trained four ML algorithms, namely Random Forests (RF), a Multilayer perceptron (MLP), Gradient Boosting (GB) and Naive Bayes (NB). We also trained three DL algorithms, namely: Graph-convolution Network (GCN) [47], Message-Passing Neural Network (MPNN) [40] and ChemBERTa [48]. We used a 80/20 train-test split and random splitting was used based on previously published results [17]. We first ran a series of simulations to assess the impact of balancing methods on the above mentioned algorithms, when trained on highly imbalanced datasets issued from HTS on the pathogens of interest (*Leishmania* parasites and Coronaviruses). We used four balancing methods. Two of them are based on random sampling, namely: Random Oversampling (ROS) and Random Undersampling (RUS). The remaining two are the Synthetic Minority Over-sampling TEchnique (SMOTE) [49] and the NearMiss technique [50]. Additionally, we performed the models training on versions of the HTS datasets that are enriched with literature-issued content of *CidalsDB*. Performances were assessed through multiple metrics detailed in the following section.

### Performance metrics

Performances of the models were assessed through different metrics that revealed their aptitude to correctly predict the activity outcome for a given molecule or to best distinguish both active and inactive classes from each other. All metrics can be calculated based on the confusion matrix, which is a table that reports the actual activity of a molecule versus its predicted activity (Table 5), resulting in four counts: True Positives (TP), the False Positive (FP), True Negatives (TN) and False Negatives (FN).

**Table 5:** The confusion matrix

	Actual Positive	Actual Negative
Predicted Positive	TP	FP
Predicted Negative	FN	TN

#### Precision

Precision is the ratio of TPs to the sum of TPs and FPs. It measures the accuracy of positive predictions.

$$\begin{aligned} Precision = \frac{TP}{TP+FP} \end{aligned}$$

#### Recall

Recall is the ratio of TPs to the sum of TPs and FNs. It measures the ability of the model to correctly identify positive instances.

$$\begin{aligned} Recall = \frac{TP}{TP+FN} \end{aligned}$$

#### F1-score

The F1-score is a common metric used in classification tasks, which combines precision and recall into a single value.

$$\begin{aligned} F1-score = \frac{2*Precision*Recall}{Precision+Recall} \end{aligned}$$

#### Balanced accuracy

Balanced accuracy is the arithmetic mean of the True Positive Rate (TPR) and the True Negative Rate (TNR). It is highly used when dealing with imbalanced data, to replace the Accuracy metric.

$$\begin{aligned} Balanced \, accuracy = \frac{1}{2} (\frac{TP}{TP + FN} + \frac{TN}{TN + FP}) \end{aligned}$$

#### ROC-AUC

The ROC-AUC score is a performance metric that quantifies the overall ability of a model to discriminate between positive and negative instances by plotting the True Positive Rate (TPR) against the False Positive Rate (FPR) at various threshold settings. It is calculated as the Area Under the Receiver Operating Characteristic Curve (ROC-AUC).

#### The Matthews correlation coefficient

MCC is a coefficient that best reflects whether a binary classifier has been successfully distinguishing both classes [51].

$$\begin{aligned} MCC = \frac{{TP} \times {TN} - {FP} \times {FN}}{\sqrt{({TP} + {FP})({TP} + {FN})({TN} +{FP})({TN} + {FN})}} \end{aligned}$$

### Distance metrics

There exists a diverse range of similarity or distance coefficients utilized in molecular structure similarity measurement. These metrics provide different perspectives on the similarity or dissimilarity between chemical compounds [52].

#### Tanimoto

Tanimoto distance is a simple yet powerful metric. It is defined as the ratio of the intersection of the sets to the union of the sets, and is calculated as follows:

$$\begin{aligned}Tanimoto(A, B) = \frac{C}{A + B - C}\end{aligned}$$

#### Dice

The Dice distance, also known as the Dice coefficient, is a popular similarity measure used in DD. It is closely related to the Tanimoto coefficient and quantifies the degree of overlap between two sets of molecular features, such as fingerprints or descriptors, as follows:

$$\begin{aligned}Dice(A, B) = 1 - \frac{2C}{A + B}\end{aligned}$$

#### Cosine

Cosine similarity is widely used to assess the similarity between chemical compounds. It calculates the cosine of the angle between two vectors, making it suitable for comparing molecular fingerprints, descriptors, or other numerical representations of compounds, through the following formula:

$$\begin{aligned} Cosine(A,B) = \frac{{\textbf{A} \cdot \textbf{B}}}{{\Vert \textbf{A}\Vert \Vert \textbf{B}\Vert }} \end{aligned}$$

#### Tversky

The Tversky measure is particularly useful for comparing two sets or groups of elements generalizing the Jaccard index and the Dice coefficient. Tversky measure offers flexibility in capturing different aspects of set similarity, and is defined as follows:

$$\begin{aligned} Tversky(A, B) = \frac{|A \cap B|}{|A \cap B| + \alpha |A \setminus B| + \beta |B \setminus A|} \end{aligned}$$

## Supplementary Information

Supplementary Material 1

Supplementary Material 2

Supplementary Material 3

Supplementary Material 4

Supplementary Material 5

## Data Availability

All data generated and used through the present publication can be accessed through our Github repository: https://github.com/Harigua/CidalsDB.

## Abbreviations

ADMET: Absorption, Distribution, Metabolism, Excretion, and Toxicity
AI: Artificial Intelligence
AID: Assay Identification Number
ANNs: Artificial Neural Networks
CADD: Computer-Aided Drug Discovery and Design
CID: Compound Identifier
CidalsDB: Cidals Data-Base
COVID-19: Coronavirus Disease 2019
CSV format: Comma-Separated Values
DD: Drug Discovery
DL: Deep learning
ECFPs: Extended Connectivity Fingerprints
EL: Ensemble Learning
FN: False Negative
FP: False Positive
FPR: False Positive Rate
FPs: Fingerprints
GB: Gradient Boosting
GCN: Graph Convolution Network
HTS: High-Throughput Screening
*L*.*major*: $$Leishmania\,major$$
MCC: Matthews Correlation Coefficient
MERS: Middle East Respiratory Syndrome
ML: Machine Learning
MLP: Multi-Layer Perceptron
MNA: Multilevel Neighborhoods of Atoms
MPNN: Message Passing Neural Network
NB: Naive Bayes
NTDs: Neglected Tropical Diseases
PDB: Protein Data Bank
PLpro: Papain-Like Protease
RF: Random Forest
ROC-AUC: Receiver Operating Characteristic Area Under the Curve
ROS: Random Over-Sampling
RUS: Random Under-Sampling
SAR: Structure-Activity Relationship
SARS-CoV-2: Severe Acute Respiratory Syndrome Coronavirus 2
SDF: Structure Data File
SMILES: Simplified Molecular-Input Line Entry System
SMOTE: Synthetic Minority Over-sampling Technique
TCMBank: Traditional Chinese Medicine Bank
TN: True Negative
TNR: True Negative Rate
TP: True Positive
TPR: True Positive Rate
3CLpro: 3 Cysteine-Like Protease

## Code availability

All code generated through the present work and underlying the published results can be accessed through our Github repository: https://github.com/Harigua/CidalsDB.

## References

[1] Sayers EW, Bolton EE, Brister JR, Canese K, Chan J, Comeau DC, Connor R, Funk K, Kelly C, Kim S (2022) Database resources of the national center for biotechnology information. Nucleic Acids Res 50(D1):20–26 10.1093/nar/gkab1112 PMC8728269 34850941

[2] Bairoch A, Apweiler R, Wu CH, Barker WC, Boeckmann B, Ferro S, Gasteiger E, Huang H, Lopez R, Magrane M (2005) The universal protein resource (uniprot). Nucleic Acids Res 33(suppl–1):154–159 10.1093/nar/gki070 PMC540024 15608167

[3] Irwin JJ, Shoichet BK (2005) Zinc- a free database of commercially available compounds for virtual screening. J Chem Inf Model 45(1):177–182 15667143 10.1021/ci049714 PMC1360656

[4] Ayers M (2012) Chemspider: the free chemical database. Ref Rev 26(7):45–46

[5] Matos P, Dekker A, Ennis M, Hastings J, Haug K, Turner S, Steinbeck C (2010) Chebi: a chemistry ontology and database. J Cheminformat 2:1–1

[6] Wang Y, Suzek T, Zhang J, Wang J, He S, Cheng T, Shoemaker BA, Gindulyte A, Bryant SH (2014) Pubchem bioassay: 2014 update. Nucleic Acids Res 42(D1):1075–1082 10.1093/nar/gkt978 PMC3965008 24198245

[7] Kim S, Thiessen PA, Bolton EE, Chen J, Fu G, Gindulyte A, Han L, He J, He S, Shoemaker BA (2016) Pubchem substance and compound databases. Nucleic Acids Res 44(D1):1202–1213 10.1093/nar/gkv951 PMC4702940 26400175

[8] Kouranov A, Xie L, Cruz J, Chen L, Westbrook J, Bourne PE, Berman HM (2006) The rcsb pdb information portal for structural genomics. Nucleic Acids Res 34:302–305 10.1093/nar/gkj120 PMC1347482 16381872

[9] Callaway E (2024) Chemistry nobel goes to developers of alphafold ai that predicts protein structures. Nature 634(8034):525–526 39384918 10.1038/d41586-024-03214-7

[10] Spangenberg T, Burrows JN, Kowalczyk P, McDonald S, Wells TN, Willis P (2013) The open access malaria box: a drug discovery catalyst for neglected diseases. PLoS ONE 8(6):62906 10.1371/journal.pone.0062906 PMC3684613 23798988

[11] Van Voorhis WC, Adams JH, Adelfio R, Ahyong V, Akabas MH, Alano P, Alday A, Alemán Resto Y, Alsibaee A, Alzualde A (2016) Open source drug discovery with the malaria box compound collection for neglected diseases and beyond. PLoS Pathogens 12(7):1005763 10.1371/journal.ppat.1005763 PMC4965013 27467575

[12] Ogunleye AJ, Olaolu OS, Ibrahim NB, James AA (2021) Molecular docking, qsar and microscopic studies of anti-trypanosomal compounds from the pathogen box. Curr Comput Aided Drug Design 17(3):378–386 10.2174/1573409916666200722140704 32703140

[13] Vijayakumar S, Kant V, Das P (2019) Leishindb: a web-accessible resource for small molecule inhibitors against leishmania sp. Acta Tropica 190:375–379 30552881 10.1016/j.actatropica.2018.12.022

[14] Zouhir A, Souiai O, Harigua E, Cherif A, Chaalia AB, Sebei K (2023) Antipseudobase: database of antimicrobial peptides and essential oils against pseudomonas. Int J Peptide Res Ther 29(3):37

[15] Lv Q, Chen G, He H, Yang Z, Zhao L, Chen H-Y, Chen CY-C (2023) Tcmbank: bridges between the largest herbal medicines, chemical ingredients, target proteins, and associated diseases with intelligence text mining. Chem Sci 14(39):10684–10701 37829020 10.1039/d3sc02139d PMC10566508

[16] Krallinger M, Rabal O, Lourenco A, Oyarzabal J, Valencia A (2017) Information retrieval and text mining technologies for chemistry. Chem Rev 117(12):7673–7761 28475312 10.1021/acs.chemrev.6b00851

[17] Harigua-Souiai E, Heinhane MM, Abdelkrim YZ, Souiai O, Abdeljaoued-Tej I, Guizani I (2021) Deep learning algorithms achieved satisfactory predictions when trained on a novel collection of anticoronavirus molecules. Front Genet 12:744170 34912370 10.3389/fgene.2021.744170 PMC8667578

[18] Tzou PL, Tao K, Nouhin J, Rhee S-Y, Hu BD, Pai S, Parkin N, Shafer RW (2020) Coronavirus antiviral research database (cov-rdb): an online database designed to facilitate comparisons between candidate anti-coronavirus compounds. Viruses 12(9):1006 32916958 10.3390/v12091006 PMC7551675

[19] Nwaka S, Besson D, Ramirez B, Maes L, Matheeussen A, Bickle Q, Mansour NR, Yousif F, Townson S, Gokool S (2011) Integrated dataset of screening hits against multiple neglected disease pathogens. PLoS Negl Trop Dis 5(12):1412 10.1371/journal.pntd.0001412 PMC3243694 22247786

[20] Venkatraman V, Colligan TH, Lesica GT, Olson DR, Gaiser J, Copeland CJ, Wheeler TJ, Roy A (2022) Drugsniffer: an open source workflow for virtually screening billions of molecules for binding affinity to protein targets. Front Pharmacol 13:874746 35559261 10.3389/fphar.2022.874746 PMC9086895

[21] Druzhilovskiy DS, Stolbov LA, Savosina PI, Pogodin PV, Filimonov DA, Veselovsky AV, Stefanisko K, Tarasova NI, Nicklaus MC, Poroikov VV (2020) Computational approaches to identify a hidden pharmacological potential in large chemical libraries. Supercomput Front Innov 7(3)

[22] Vistoli G, Manelfi C, Talarico C, Fava A, Warshel A, Tetko IV, Apostolov R, Ye Y, Latini C, Ficarelli F (2023) Mediate-molecular docking at home: turning collaborative simulations into therapeutic solutions. Expert Opin Drug Discov 18(8):821–833 37424369 10.1080/17460441.2023.2221025 PMC12404243

[23] Harigua-Souiai E, Oualha R, Souiai O, Abdeljaoued-Tej I, Guizani I (2022) Applied machine learning toward drug discovery enhancement: Leishmaniases as a case study. Bioinform Biol Insights 16:11779322221090348 35478992 10.1177/11779322221090349 PMC9036323

[24] Oualha R, Abdelkrim YZ, Guizani I, Harigua-Souiai E (2024) Approved drugs successfully repurposed against leishmania based on machine learning predictions. Front Cell Infect Microbiol 14:1403589. 10.3389/fcimb.2024.1403589 39391884 10.3389/fcimb.2024.1403589 PMC11464777

[25] Liu Z, Du J, Fang J, Yin Y, Xu G, Xie L (2019) Deepscreening: a deep learning-based screening web server for accelerating drug discovery. Database 2019:104 10.1093/database/baz104 PMC6790966 31608949

[26] CAS: CAS COVID-19 Antiviral Candidate Compounds Dataset. https://www.cas.org/covid-19-sar-dataset. [Online; No longer accessible as of October 2024] (2020)

[27] Knox C, Wilson M, Klinger CM, Franklin M, Oler E, Wilson A, Pon A, Cox J, Chin NE, Strawbridge SA (2024) Drugbank 6.0: the drugbank knowledgebase for 2024. Nucleic Acids Res 52(D1):1265–1275 10.1093/nar/gkad976 PMC10767804 37953279

[28] Martin H-J, Melo-Filho CC, Korn D, Eastman RT, Rai G, Simeonov A, Zakharov AV, Muratov E, Tropsha A (2022) Small molecule antiviral compound collection (smacc): a database to support the discovery of broad-spectrum antiviral drug molecules. bioRxiv 10.1016/j.antiviral.2023.105620 PMC11069349 37169224

[29] Bess A, Berglind F, Mukhopadhyay S, Brylinski M, Alvin C, Fattah F, Wasan KM (2023) Identification of oral therapeutics using an ai platform against the virus responsible for covid-19, sars-cov-2. Front Pharmacol 14:1297924 38186640 10.3389/fphar.2023.1297924 PMC10770831

[30] Akash S, Bibi S, Biswas P, Mukerjee N, Khan DA, Hasan MN, Sultana NA, Hosen ME, Jardan YAB, Nafidi H-A (2023) Revolutionizing anti-cancer drug discovery against breast cancer and lung cancer by modification of natural genistein: an advanced computational and drug design approach. Front Oncol 13:1228865 37817764 10.3389/fonc.2023.1228865 PMC10561655

[31] Sakyi PO, Broni E, Amewu RK, Miller WA III, Wilson MD, Kwofie SK (2023) Targeting leishmania donovani sterol methyltransferase for leads using pharmacophore modeling and computational molecular mechanics studies. Inform Med Unlocked 37:101162

[32] Lagunin A, Stepanchikova A, Filimonov D, Poroikov V (2000) Pass: prediction of activity spectra for biologically active substances. Bioinformatics 16(8):747–748 11099264 10.1093/bioinformatics/16.8.747

[33] Filimonov D, Poroikov V, Borodina Y, Gloriozova T (1999) Chemical similarity assessment through multilevel neighborhoods of atoms: definition and comparison with the other descriptors. J Chem Inf Comput Sci 39(4):666–670

[34] Lagunin A, Filimonov D, Poroikov V (2010) Multi-targeted natural products evaluation based on biological activity prediction with pass. Curr Pharm Design 16(15):1703–1717 10.2174/138161210791164063 20222853

[35] Swanson K, Walther P, Leitz J, Mukherjee S, Wu JC, Shivnaraine RV, Zou J (2024) Admet-ai: a machine learning admet platform for evaluation of large-scale chemical libraries. Bioinformatics 40(7):416 10.1093/bioinformatics/btae416 PMC11226862 38913862

[36] Myung Y, Sá AG, Ascher DB (2024) Deep-pk: deep learning for small molecule pharmacokinetic and toxicity prediction. Nucleic Acids Res. 10.1093/nar/gkae254 38634808 10.1093/nar/gkae254 PMC11223837

[37] Daina A, Michielin O, Zoete V (2017) Swissadme: a free web tool to evaluate pharmacokinetics, drug-likeness and medicinal chemistry friendliness of small molecules. Sci Rep 7(1):42717 28256516 10.1038/srep42717 PMC5335600

[38] Tian H, Ketkar R, Tao P (2022) Admetboost: a web server for accurate admet prediction. J Mol Model 28(12):408 36454321 10.1007/s00894-022-05373-8 PMC9903341

[39] Xiong G, Wu Z, Yi J, Fu L, Yang Z, Hsieh C, Yin M, Zeng X, Wu C, Lu A (2021) Admetlab 2.0: an integrated online platform for accurate and comprehensive predictions of admet properties. Nucleic acids research 49(W1):5–14 10.1093/nar/gkab255 PMC8262709 33893803

[40] Dai H, Dai B, Song L (2016) Discriminative embeddings of latent variable models for structured data. In: International Conference on Machine Learning, pp. 2702–2711. PMLR

[41] He H, Bai Y, Garcia, EA, Li S (2008) Adasyn: Adaptive synthetic sampling approach for imbalanced learning. In: the International Joint Conference on Neural Networks, IJCNN 2008, Part of IEEE World Congress on Computational Intelligence, WCCI 2008, IEEE, pp. 1322–1328

[42] Korkmaz S (2020) Deep learning-based imbalanced data classification for drug discovery. J Chem Inf Model 60(9):4180–4190 32573225 10.1021/acs.jcim.9b01162

[43] Setting up Mysql and Phpmyadmin, pp. 261–283. Apress, Berkeley, CA (2007). 10.1007/978-1-4302-0275-2_10

[44] Landrum G (2010) RDkit. https://www.rdkit.org/

[45] Democratizing Deep-Learning for Drug Discovery (2016) Quantum Chemistry. Materials Science and Biology, GitHub

[46] Wolf T, Debut L, Sanh V, Chaumond J, Delangue C, Moi A, Cistac P, Rault T, Louf R, Funtowicz M (2020) Transformers: State-of-the-art natural language processing. In: Proceedings of the 2020 Conference on Empirical Methods in Natural Language Processing: System Demonstrations, pp. 38–45

[47] Kipf TN, Welling M (2017) Semi-supervised classification with graph convolutional networks. ICLR

[48] Chithrananda S, Grand G, Ramsundar B (2020) Chemberta: large-scale self-supervised pretraining for molecular property prediction. arXiv preprint arXiv:2010.09885

[49] Chawla NV, Bowyer KW, Hall LO, Kegelmeyer WP (2002) Smote: synthetic minority over-sampling technique. J Artif Intell Res 16:321–357

[50] Zhang J, Mani I (2003) Knn approach to unbalanced data distributions: A case study involving information extraction. In: Proceedings of the ICML’2003 Workshop on Learning from Imbalanced Datasets, vol. 126

[51] Chicco D, Jurman G (2020) The advantages of the Matthews correlation coefficient (mcc) over f1 score and accuracy in binary classification evaluation. BMC Genomics 21(1):1–13 10.1186/s12864-019-6413-7 PMC6941312 31898477

[52] Choi S-S, Cha S-H, Tappert CC (2010) A survey of binary similarity and distance measures. J Syst Cybern Inform 8(1):43–48

