# Supplementary material for: cidalsDB: an AI-empowered platform for anti-pathogen therapeutics research
Source: J Cheminform. 2024 Nov 28;16:134. doi: 10.1186/s13321-024-00929-7 (PMC11605991; doi:10.1186/s13321-024-00929-7)
Supplement: Supplementary file 1 — Supplementary Material 1 [file 13321_2024_929_MOESM1_ESM.pdf]

CidalsDB: An AI-empowered platform for anti-pathogen therapeutics research

Authors: Emna Harigua-Souiai*[(1)], Ons Masmoudi[(1)], Samer Makni[(1)], Rafeh Oualha[(1)], Yosser Z. Abdelkrim[(1)], Sara Hamdi[(1)], Oussama Souiai[(2)], Ikram Guizani[(1)]
(*) corresponding author: emna.harigua@pasteur.utm.tn

Supplementary Figures:

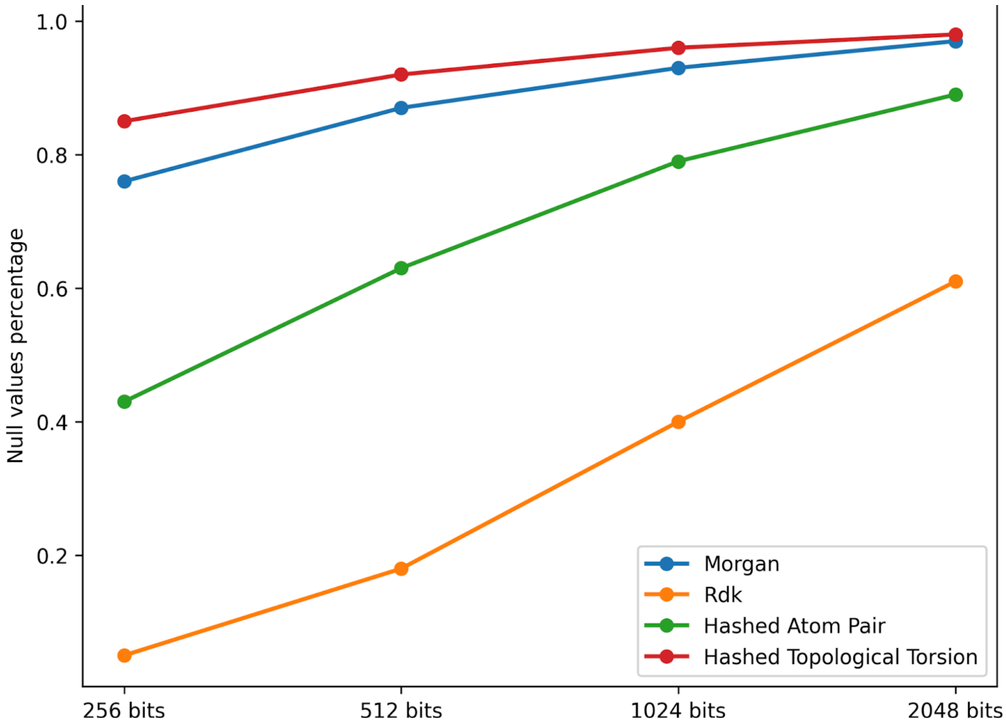

Figure S1: Variation of the percentage of zeros with the vector size of the different fingerprints (FPs).

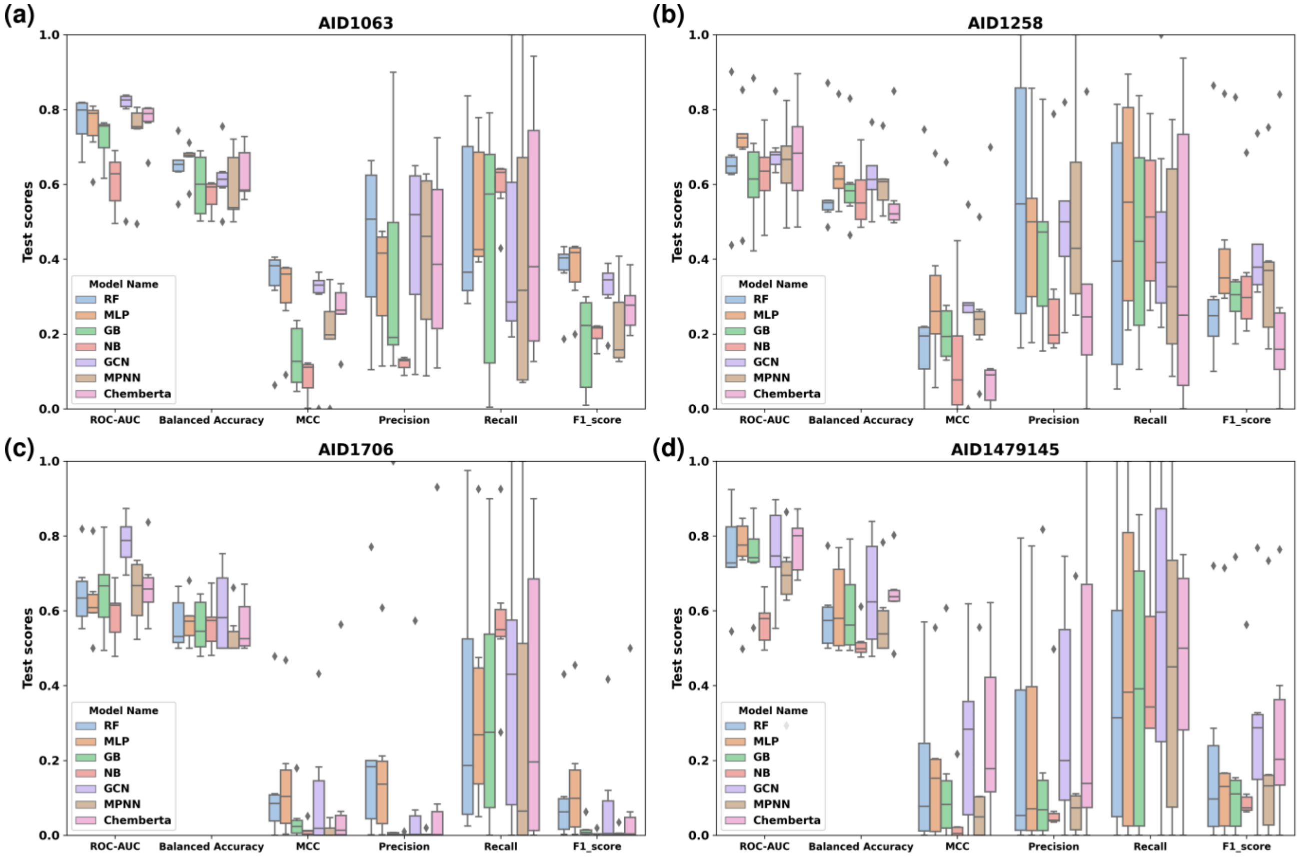

Figure S2: Algorithms' performances achieved by the different models (4 ML and 3 DL), when trained on the 3 versions of each bioassay (Original, RUS, ROS and Enriched). (a) Each ML model's performance when trained on all versions of the AID1063 dataset. (b) Each ML model's performance when trained on all versions of the AID1258 dataset. (c) Each ML model's performance when trained on all versions of the AID1706 dataset. (d) Each ML model's performance when trained on all versions of the AID1479145 dataset.

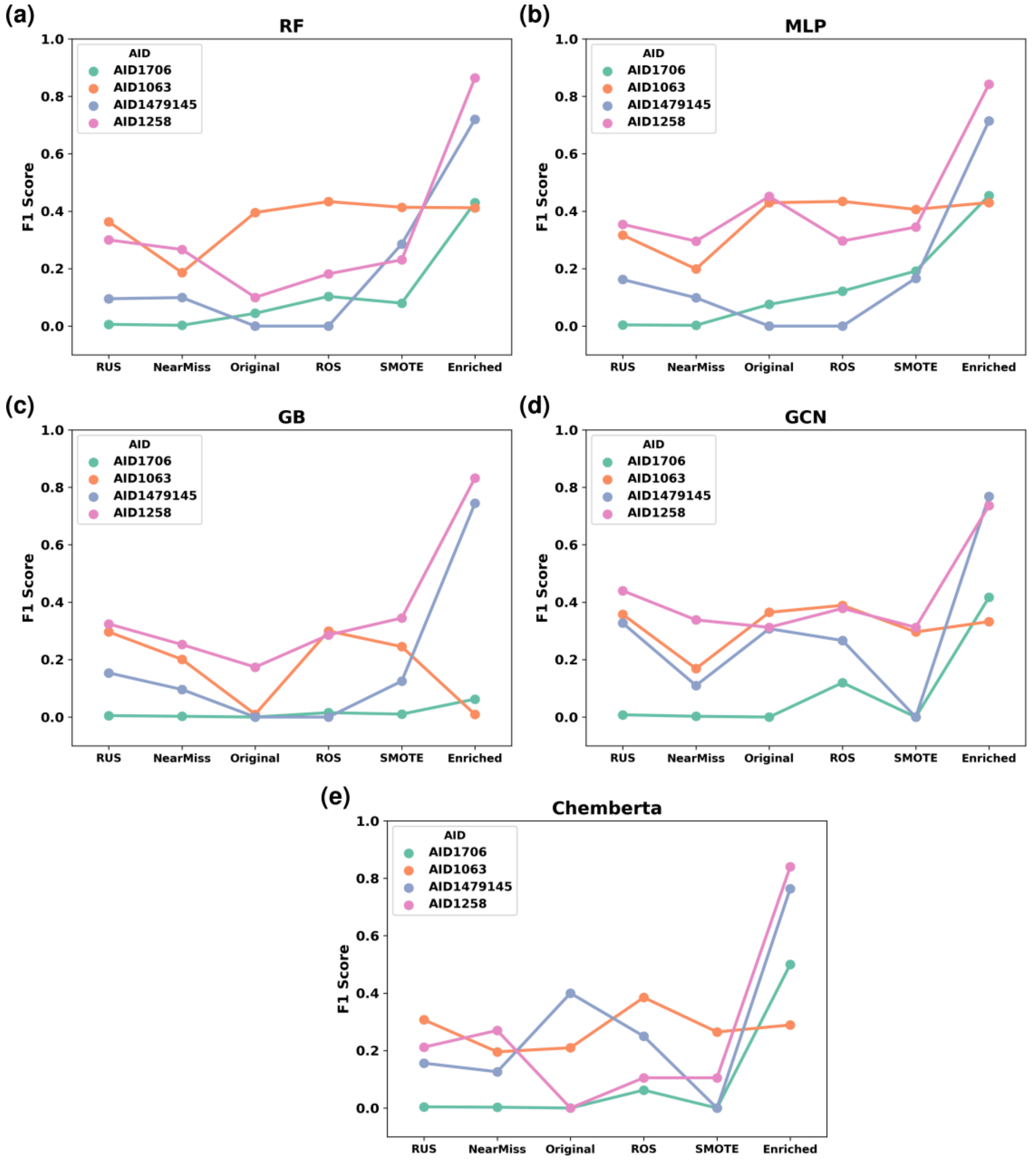

Figure S3: Effects of the different balancing methods and the enrichment approach on the different datasets per model based on F1-score (a) RF performances when trained on the different variations of the different datasets. (b) MLP performances when trained on the different variations of the different datasets. (c) GB performances when trained on the different variations of the different datasets. (d) GCN performances when trained

on the different variations of the different datasets. (e) ChemBERTa performances when trained on the different variations of the different datasets.

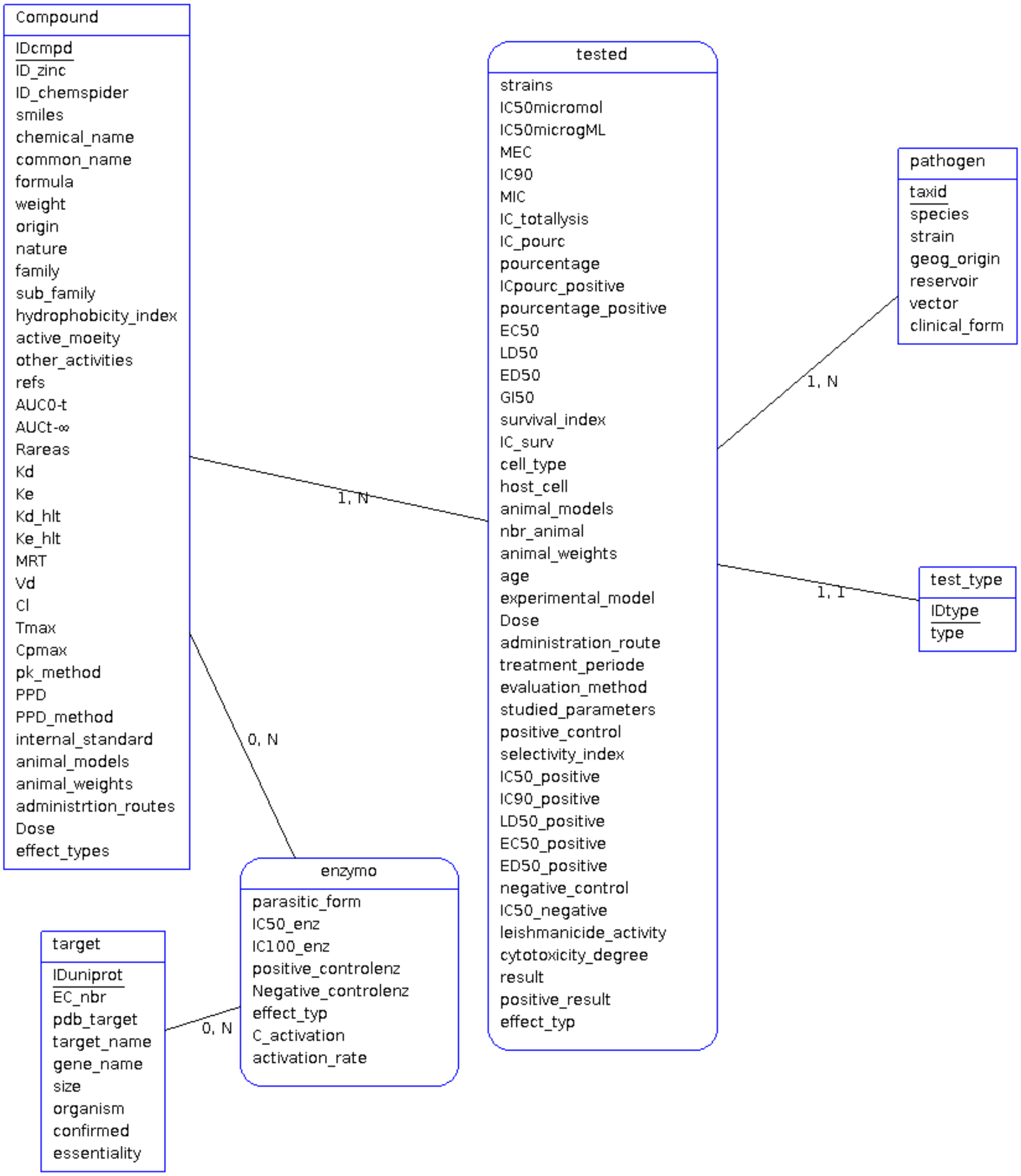

Figure S4: Conceptual Data Model (CMD) of the database CidalsDB.
