# Supplementary material for: cidalsDB: an AI-empowered platform for anti-pathogen therapeutics research
Source: J Cheminform. 2024 Nov 28;16:134. doi: 10.1186/s13321-024-00929-7 (PMC11605991; doi:10.1186/s13321-024-00929-7)
Supplement: Supplementary file 3 — Supplementary Material 3 [file 13321_2024_929_MOESM3_ESM.pdf]

CidalsDB: An AI-empowered platform for anti-pathogen therapeutics research

Authors: Emna Harigua-Souiai*(1), Ons Masmoudi(1), Samer Makni(1), Rafeh Oualha(1), Yosser Z. Abdelkrim(1), Sara Hamdi(1), Oussama Souiai(2), Ikram Guizani(1)
(*) corresponding author: emna.harigua@pasteur.utm.tn

Supplementary Table: Results of all simulations (Original, Resampled & Enriched) of the *Leishmania* (AID1063 & AID1258) and the Coronaviruses (AID1706 & AID1479145) bioassays on the performances of the ML and DL algorithms in molecule classification.

Leishmania

AID 1063

Data AID 1063	Model	Accuracy	Balanced Accuracy	ROC-AUC	MCC	Precision	Recall	F1 score
Original	RF	0.92	0.63	0.82	0.40	0.66	0.28	0.40
	MLP	0.90	0.67	0.80	0.38	0.47	0.39	0.43
	NB	0.57	0.60	0.64	0.12	0.13	0.64	0.22
	GB	0.91	0.50	0.76	0.06	0.90	0	0.01
	GCN	0.92	0.58	0.83	0.30	0.67	0.16	0.26
	MPNN	0.91	0.53	0.75	0.19	0.61	0.07	0.13
	ChemBERTa	0.91	0.56	0.77	0.25	0.62	0.13	0.21

Dataset	Model	Accuracy	Balanced Accuracy	ROC-AUC	MCC	Precision	Recall	F1 score
AID1063 Enriched with Cidals	RF	0.91	0.64	0.81	0.40	0.65	0.30	0.41
	MLP	0.89	0.67	0.80	0.37	0.46	0.40	0.43
	NB	0.60	0.59	0.62	0.11	0.13	0.56	0.22
	GB	0.90	0.50	0.75	0.04	0.6	0.004	0.009
	GCN	0.91	0.61	0.83	0.35	0.64	0.24	0.35
	MPNN	0.90	0.53	0.74	0.19	0.65	0.07	0.12
	ChemBERTa	0.91	0.59	0.80	0.33	0.72	0.18	0.29

Data AID1063	Model	Accuracy	Balanced Accuracy	ROC-AUC	MCC	Precision	Recall	F1 score
ROS	RF	0.91	0.66	0.81	0.40	0.54	0.36	0.43
	MLP	0.90	0.68	0.81	0.38	0.44	0.42	0.43
	NB	0.54	0.60	0.66	0.11	0.13	0.63	0.21
	GB	0.70	0.69	0.76	0.23	0.19	0.67	0.30
	GCN	0.80	0.75	0.83	0.34	0.27	0.70	0.39
	MPNN	0.85	0.71	0.80	0.34	0.32	0.54	0.40
	ChemBERTa	0.83	0.72	0.80	0.32	0.29	0.58	0.38

Data AID1063	Model	Accuracy	Balanced Accuracy	ROC-AUC	MCC	Precision	Recall	F1 score
SMOTE	RF	0.90	0.66	0.78	0.37	0.47	0.37	0.41
	MLP	0.89	0.68	0.78	0.34	0.39	0.42	0.41
	NB	0.56	0.50	0.49	0.001	0.09	0.43	0.18
	GB	0.74	0.62	0.68	0.16	0.16	0.47	0.24
	GCN	0.91	0.59	0.82	0.32	0.65	0.19	0.30
	MPNN	0.91	0.53	0.75	0.19	0.60	0.07	0.13
	ChemBERTa	0.90	0.58	0.76	0.26	0.48	0.18	0.26

Data AID1063	Model	Accuracy	Balanced Accuracy	ROC-AUC	MCC	Precision	Recall	F1 score
RUS	RF	0.76	0.74	0.72	0.31	0.24	0.81	0.36
	MLP	0.71	0.71	0.71	0.26	0.20	0.77	0.32
	NB	0.53	0.60	0.69	0.12	0.13	0.64	0.22
	GB	0.69	0.69	0.75	0.23	0.19	0.68	0.30
	GCN	0.90	0.63	0.80	0.30	0.42	0.31	0.36
	MPNN	0.72	0.72	0.80	0.28	0.21	0.71	0.32
	ChemBERTa	0.67	0.73	0.80	0.27	0.19	0.80	0.31

Data AID1063	Model	Accuracy	Balanced Accuracy	ROC-AUC	MCC	Precision	Recall	F1 score
NearMiss	RF	0.31	0.55	0.66	0.063	0.10	0.84	0.19
	MLP	0.41	0.57	0.60	0.009	0.11	0.71	0.20
	NB	0.45	0.53	0.53	0.04	0.10	0.63	0.18
	GB	0.40	0.58	0.61	0.09	0.11	0.79	0.20
	GCN	0.09	0.5	0.50	0	0.09	1	0.17
	MPNN	0.09	0.5	0.49	0	0.08	1	0.16
	ChemBERTa	0.29	0.58	0.65	0.11	0.11	0.94	0.19

AID 1258

Data AID1258	Model	Accuracy	Balanced Accuracy	ROC-AUC	MCC	Precision	Recall	F1 score
Original	**RF**	0.84	0.53	0.68	0.21	1	0.05	0.10
	**MLP**	0.85	0.66	0.74	0.38	0.58	0.37	0.45
	**NB**	0.75	0.62	0.68	0.22	0.32	0.42	0.36
	**GB**	0.83	0.54	0.62	0.17	0.50	0.11	0.17
	**GCN**	0.81	0.59	0.68	0.26	0.56	0.22	0.31
	**MPNN**	0.78	0.60	0.71	0.23	0.43	0.30	0.36
	**ChemBERTa**	0.84	0.5	0.57	0	0	0	0

Dataset	Model	Accuracy	Balanced Accuracy	ROC-AUC	MCC	Precision	Recall	F1 score
AID1258 Enriched with Cidals	**RF**	0.87	0.87	0.90	0.74	0.92	0.81	0.86
	**MLP**	0.84	0.84	0.85	0.68	0.86	0.83	0.84
	**NB**	0.71	0.71	0.77	0.44	0.78	0.60	0.68
	**GB**	0.82	0.82	0.88	0.65	0.82	0.83	0.83
	**GCN**	0.77	0.76	0.85	0.54	0.82	0.67	0.73
	**MPNN**	0.75	0.76	0.82	0.51	0.73	0.77	0.75
	**ChemBERTa**	0.88	0.88	0.93	0.76	0.93	0.84	0.88

Data AID1258	Model	Accuracy	Balanced Accuracy	ROC-AUC	MCC	Precision	Recall	F1 score
ROS	RF	0.84	0.54	0.64	0.22	0.66	0.10	0.18
	MLP	0.83	0.58	0.73	0.24	0.5	0.21	0.30
	NB	0.64	0.51	0.61	0.023	0.18	0.31	0.23
	GB	0.82	0.58	0.71	0.22	0.44	0.21	0.28
	GCN	0.80	0.61	0.65	0.28	0.5	0.30	0.38
	MPNN	0.77	0.61	0.67	0.24	0.42	0.35	0.38
	ChemBERTa	0.85	0.52	0.74	0.09	0.33	0.062	0.10

Data AID1258	Model	Accuracy	Balanced Accuracy	ROC-AUC	MCC	Precision	Recall	F1 score
SMOTE	RF	0.82	0.55	0.63	0.18	0.42	0.16	0.23
	MLP	0.83	0.60	0.69	0.28	0.5	0.26	0.34
	NB	0.66	0.50	0.57	0.006	0.17	0.26	0.20
	GB	0.83	0.60	0.61	0.27	0.5	0.26	0.34
	GCN	0.80	0.59	0.70	0.26	0.55	0.21	0.3125
	MPNN	0.81	0.54	0.58	0.26	1	0.09	0.16
	ChemBERTa	0.85	0.52	0.57	0.09	0.33	0.063	0.10

Data AID1258	Model	Accuracy	Balanced Accuracy	ROC-AUC	MCC	Precision	Recall	F1 score
RUS	RF	0.50	0.55	0.65	0.08	0.19	0.63	0.3
	MLP	0.54	0.62	0.71	0.18	0.23	0.73	0.35
	NB	0.52	0.59	0.65	0.13	0.21	0.68	0.32
	GB	0.56	0.58	0.55	0.13	0.22	0.63	0.32
	GCN	0.75	0.65	0.68	0.28	0.40	0.48	0.44
	MPNN	0.54	0.61	0.66	0.18	0.27	0.73	0.39
	ChemBERTa	0.54	0.50	0.48	-0.004	0.14	0.4375	0.21

Data AID1258	Model	Accuracy	Balanced Accuracy	ROC-AUC	MCC	Precision	Recall	F1 score
NearMiss	RF	0.32	0.48	0.43	-0.02	0.16	0.73	0.26
	MLP	0.28	0.52	0.48	0.05	0.17	0.89	0.29
	NB	0.28	0.48	0.46	-0.02	0.16	0.79	0.27
	GB	0.32	0.46	0.42	-0.06	0.15	0.68	0.25
	GCN	0.20	0.5	0.63	0	0.20	1	0.33
	MPNN	0.74	0.51	0.48	0.04	0.25	0.13	0.17
	ChemBERTa	0.28	0.55	0.63	0.11	0.16	0.93	0.27

<u>COVID :</u>

AID1706

Dataset AID1706	Model	Accuracy	Balanced Accuracy	ROC-AUC	MCC	Precision	Recall	F1 score
Original	**RF**	0.99	0.51	0.62	0.07	0.2	0.025	0.04
	**MLP**	0.99	0.52	0.60	0.08	0.15	0.05	0.07
	**NB**	0.64	0.58	0.62	0.013	0.002	0.525	0.004
	**GB**	0.99	0.5	0.66	0	0	0	0
	**GCN**	0.99	0.5	0.73	0	0	0	0
	**MPNN**	0.99	0.5	0.61	0	0	0	0
	**ChemBERTa**	0.99	0.5	0.65	0	0	0	0

Dataset	Model	Accuracy	Balanced Accuracy	ROC-AUC	MCC	Precision	Recall	F1 score
AID1706 Enriched with Cidals	**RF**	0.99	0.65	0.82	0.47	0.77	0.30	0.43
	**MLP**	0.99	0.68	0.81	0.47	0.61	0.36	0.45
	**NB**	0.72	0.67	0.69	0.05	0.01	0.62	0.02
	**GB**	0.99	0.51	0.82	0.18	1	0.032	0.062
	**GCN**	0.99	0.66	0.87	0.43	0.57	0.33	0.42
	**MPNN**	0.99	0.50	0.73	0	0	0	0
	**ChemBERTa**	0.99	0.67	0.84	0.56	0.93	0.34	0.5

Dataset AID1706	Model	Accuracy	Balanced Accuracy	ROC-AUC	MCC	Precision	Recall	F1 score
ROS	**RF**	0.99	0.54	0.65	0.11	0.16	0.075	0.10
	**MLP**	0.99	0.56	0.65	0.12	0.12	0.125	0.12
	**NB**	0.61	0.58	0.62	0.012	0.002	0.55	0.004
	**GB**	0.94	0.64	0.67	0.044	0.008	0.35	0.015
	**GCN**	0.97	0.75	0.83	0.18	0.06	0.53	0.12
	**MPNN**	0.99	0.56	0.72	0.04	0.02	0.13	0.03
	**ChemBERTa**	0.99	0.52	0.55	0.06	0.08	0.05	0.063

Dataset AID1706	Model	Accuracy	Balanced Accuracy	ROC-AUC	MCC	Precision	Recall	F1 score
SMOTE	**RF**	0.99	0.52	0.57	0.1	0.2	0.05	0.08
	**MLP**	0.99	0.59	0.59	0.19	0.21	0.175	0.19
	**NB**	0.68	0.48	0.48	-0.003	0.001	0.275	0.002
	**GB**	0.94	0.57	0.55	0.02	0.005	0.2	0.01
	**GCN**	0.99	0.5	0.80	0	0	0	0
	**MPNN**	0.99	0.5	0.58	0	0	0	0
	**ChemBERTa**	0.99	0.5	0.66	0	0	0	0

Dataset AID1706	Model	Accuracy	Balanced Accuracy	ROC-AUC	MCC	Precision	Recall	F1 score
RUS	RF	0.72	0.66	0.69	0.03	0.003	0.6	0.006
	MLP	0.69	0.58	0.62	0.013	0.002	0.475	0.004
	NB	0.58	0.56	0.61	0.01	0.002	0.55	0.004
	GB	0.68	0.64	0.70	0.021	0.002	0.6	0.005
	GCN	0.80	0.70	0.76	0.03	0.004	0.59	0.008
	MPNN	0.68	0.66	0.75	0.025	0.003	0.64	0.005
	ChemBERTa	0.48	0.64	0.70	0.02	0.002	0.8	0.004

Dataset AID1706	Model	Accuracy	Balanced Accuracy	ROC-AUC	MCC	Precision	Recall	F1 score
NearMiss	RF	0.03	0.50	0.55	0.006	0.001	0.975	0.002
	MLP	0.075	0.50	0.50	0.004	0.0013	0.925	0.003
	NB	0.08	0.50	0.52	0.0007	0.0013	0.925	0.003
	GB	0.06	0.48	0.49	-0.007	0.0013	0.9	0.002
	GCN	0.001	0.5	0.69	0.0003	0.001	1	0.002
	MPNN	0.001	0.5	0.52	0	0.001	1	0.002
	ChemBERTa	0.15	0.53	0.61	0.005	0.001	0.9	0.003

AID 1479145

Dataset AID1479145	Model	Accuracy	Balanced Accuracy	ROC-AUC	MCC	Precision	Recall	F1 score
Original	RF	0.96	0.50	0.72	0	0	0	0
	MLP	0.95	0.50	0.78	-0.02	0	0	0
	NB	0.68	0.50	0.57	0	0.04	0.3	0.06
	GB	0.96	0.50	0.74	0	0	0	0
	GCN	0.95	0.60	0.72	0.35	0.66	0.20	0.31
	MPNN	0.94	0.5	0.63	0	0	0	0
	ChemBERTa	0.96	0.62	0.81	0.50	1	0.25	0.4

Dataset AID1479145	Model	Accuracy	Balanced Accuracy	ROC-AUC	MCC	Precision	Recall	F1 score
AID1479145 Enriched with Cidals	RF	0.80	0.77	0.85	0.57	0.79	0.66	0.72
	MLP	0.79	0.77	0.84	0.55	0.77	0.66	0.71
	NB	0.60	0.61	0.66	0.22	0.5	0.64	0.56
	GB	0.81	0.79	0.87	0.60	0.82	0.68	0.74
	GCN	0.82	0.81	0.88	0.62	0.74	0.79	0.77
	MPNN	0.78	0.78	0.86	0.55	0.69	0.78	0.73
	ChemBERTa	0.82	0.80	0.87	0.62	0.83	0.71	0.76

Dataset AID1479145	Model	Accuracy	Balanced Accuracy	ROC-AUC	MCC	Precision	Recall	F1 score
ROS	**RF**	0.95	0.6	0.83	0.44	1	0.2	0.33
	**MLP**	0.95	0.55	0.80	0.30	1	0.1	0.18
	**NB**	0.67	0.49	0.59	-0.01	0.04	0.28	0.06
	**GB**	0.95	0.6	0.84	0.43	1	0.2	0.33
	**GCN**	0.87	0.65	0.71	0.22	0.2	0.4	0.26
	**MPNN**	0.82	0.57	0.74	0.1	0.1	0.3	0.16
	**ChemBERTa**	0.89	0.65	0.68	0.21	0.18	0.375	0.25

Dataset AID1479145	Model	Accuracy	Balanced Accuracy	ROC-AUC	MCC	Precision	Recall	F1 score
SMOTE	**RF**	0.94	0.59	0.73	0.29	0.5	0.2	0.28
	**MLP**	0.94	0.55	0.77	0.20	0.5	0.1	0.16
	**NB**	0.62	0.51	0.49	0.02	0.06	0.4	0.11
	**GB**	0.92	0.53	0.72	0.12	0.16	0.1	0.125
	**GCN**	0.90	0.48	0.77	-0.05	0	0	0
	**MPNN**	0.94	0.5	0.70	0	0	0	0
	**ChemBERTa**	0.92	0.48	0.82	-0.04	0	0	0

Dataset AID1479145	Model	Accuracy	Balanced Accuracy	ROC-AUC	MCC	Precision	Recall	F1 score
RUS	**RF**	0.67	0.55	0.72	0.04	0.05	0.43	0.09
	**MLP**	0.64	0.74	0.86	0.20	0.09	0.85	0.16
	**NB**	0.65	0.47	0.59	-0.02	0.03	0.28	0.0625
	**GB**	0.68	0.70	0.80	0.16	0.09	0.71	0.15
	**GCN**	0.78	0.84	0.90	0.36	0.2	0.9	0.33
	**MPNN**	0.61	0.61	0.69	0.10	0.09	0.6	0.15
	**ChemBERTa**	0.68	0.66	0.79	0.14	0.09	0.625	0.15

Dataset AID1479145	Model	Accuracy	Balanced Accuracy	ROC-AUC	MCC	Precision	Recall	F1 score
NearMiss	**RF**	0.26	0.61	0.54	0.11	0.05	1	0.1
	**MLP**	0.25	0.61	0.50	0.11	0.05	1	0.1
	**NB**	0.05	0.50	0.50	0.02	0.04	1	0.08
	**GB**	0.34	0.59	0.55	0.07	0.05	0.85	0.09
	**GCN**	0.06	0.5	0.55	0	0.06	1	0.11
	**MPNN**	0.06	0.5	0.29	0	0.06	1	0.11
	**ChemBERTa**	0.52	0.63	0.68	0..10	0.07	0.75	0.13
