# Supplementary material for: cidalsDB: an AI-empowered platform for anti-pathogen therapeutics research
Source: J Cheminform. 2024 Nov 28;16:134. doi: 10.1186/s13321-024-00929-7 (PMC11605991; doi:10.1186/s13321-024-00929-7)
Supplement: Supplementary file 4 — Supplementary Material 4 [file 13321_2024_929_MOESM4_ESM.pdf]

CidalsDB: An AI-empowered platform for anti-pathogen therapeutics research

Authors: Emna Harigua-Souiai*(1), Ons Masmoudi(1), Samer Makni(1), Rafeh Oualha(1), Yosser Z. Abdelkrim(1), Sara Hamdi(1), Oussama Souiai(2), Ikram Guizani(1)
(*) corresponding author: emna.harigua@pasteur.utm.tn

Supplementary Table: Impact of the enrichment of the \textit{Leishmania} (AID1258) and the \textit{Coronaviruses}(AID1479145) bioassays with \textit{CidalsDB} content on the performances of the ML and DL algorithms in molecule classification. Values of balanced accuracy, ROC-AUC, MCC, precisio recall and F1-score achieved by the different models (RF, MLP, NB, GB, GCN, MPNN and ChemBERTa) when trained on the enriched datasets are shown in the corresponding boxes.
In cases where the performances were enhanced due to the enrichment, the increase rate is shown as a positive shift between brackets. Positive shifts higher than 0.3 are shown in blue. In cases where the performances were decreased due to the enrichment, the decrease rate is shown as a negative shift between brackets (in red). Metrics values higher than 0.7 are shown in bold, except for MCC where the threshold is 0.6.

Leishmania

Dataset	Model	Accuracy	ROC- AUC	Balanced Accuracy	MCC	Precision	Recall	F1 score
AID1063 Enriched with Cidals	RF	**0.91**	**0.81**	0.64	0.40	0.65 (-0.01)	0.30 (+0.02)	**0.41** (+0.01)
	MLP	**0.89**	**0.80**	0.67	0.37	0.46 (-0.01)	0.40 (+0.01)	0.43
	NB	0.60 (+0.03)	0.62 (-0.01)	0.59 (-0.01)	0.11 (-0.01)	0.13	0.56 (-0.07)	0.22
	GB	**0.90**	0.75	0.50	0.04 (-0.02)	0.6 (-0.3)	0.004	0.009
	GCN	**0.91**	**0.83**	0.61	0.35 (-0.01)	0.64 (+0.02)	0.24 (-0.02)	0.35 (-0.01)
	MPNN	**0.90** (-0.01)	0.74 (-0.01)	0.53	0.19	0.65 (+0.04)	0.07	0.12 (-0.01)
	ChemBERTa	**0.91**	0.80 (+0.03)	0.59 (+0.03)	0.33 (+0.08)	0.72 (+0.1)	0.18 (+0.05)	0.29 (+0.08)
AID1258 Enriched with Cidals	RF	**0.87** (+0.03)	**0.90** (+0.22)	**0.87** **(+0.34)**	**0.74** **(+0.53)**	**0.92** (-0.07)	**0.81** **(+0.76)**	**0.86** **(+0.76)**
	MLP	**0.84**	**0.85** (+0.11)	**0.84** (+0.18)	**0.68** **(+0.30)**	**0.86** (+0.27)	**0.83** **(+0.46)**	**0.84** **(+0.39)**
	NB	0.71 (-0.03)	**0.77** (+0.09)	**0.71** (+0.1)	0.44 (+0.23)	**0.78** **(+0.46)**	0.60 (+0.18)	0.68 **(+0.32)**
	GB	**0.82**	**0.88** (+0.26)	**0.82** (+0.28)	**0.65** **(+0.48)**	**0.82** **(+0.32)**	**0.83** **(+0.73)**	**0.83** **(+0.66)**
	GCN	**0.77** (-0.03)	**0.85** (+0.16)	**0.76** (+0.18)	0.54 (+0.28)	**0.82** (+0.26)	0.67 **(+0.45)**	**0.73** **(+0.42)**
	MPNN	**0.75** (-0.03)	**0.82** (+0.11)	**0.76** (+0.16)	0.51 (+0.28)	**0.73** **(+0.3)**	**0.77** **(+0.47)**	**0.75** **(+0.39)**
	ChemBERTa	**0.88** (+0.04)	**0.93** **(+0.36)**	**0.88** **(+0.38)**	**0.76** **(+0.76)**	**0.93** **(+0.93)**	**0.84** **(+0.84)**	**0.88** **(+0.88)**

Coronaviruses

Dataset	Model	Accuracy	ROC- AUC	Balanced Accuracy	MCC	Precision	Recall	F1 score
**AID1706 Enriched with Cidals**	**RF**	**0.99**	**0.82** (+0.20)	**0.65** (+0.14)	0.47 **(+0.40)**	**0.77** **(+0.57)**	0.30 (+0.27)	0.43 **(+0.38)**
	**MLP**	**0.99**	**0.81** (+0.21)	**0.68** (+0.15)	0.47 **(+0.38)**	0.61 **(+0.45)**	0.36 **(+0.31)**	0.45 **(+0.38)**
	**NB**	**0.72** (+0.08)	**0.69** (+0.07)	**0.67** (+0.09)	0.05 (+0.04)	0.01	0.62 (+0.09)	0.02 (+0.01)
	**GB**	**0.99**	**0.82** (+0.16)	0.51 (+0.02)	0.18 (+0.18)	1 (+1)	0.032 (+0.032)	0.062 (+0.062)
	**GCN**	**0.99**	**0.87** (+0.14)	**0.66** (+0.16)	0.43 **(+0.43)**	0.57 **(+0.57)**	0.33 **(+0.33)**	0.42 **(+0.42)**
	**MPNN**	**0.99**	0.73 (+0.12)	0.50	0	0	0	0
	**ChemBERTa**	**0.99**	**0.84** (+0.19)	**0.67** (+0.17)	0.56 **(+0.56)**	**0.93** **(+0.93)**	0.34 (+0.34)	0.5 **(+0.5)**
**AID1479145 Enriched with Cidals**	**RF**	**0.80** (-0.16)	**0.85** (+0.27)	**0.77** (+0.14)	0.57 **(+0.57)**	**0.79** **(+0.79)**	0.66 **(+0.66)**	**0.72** **(+0.72)**
	**MLP**	**0.79** (-0.15)	**0.84** (+0.06)	**0.77** (+0.27)	0.55 **(+0.57)**	**0.77** **(+0.77)**	0.66 **(+0.66)**	**0.71** **(+0.71)**
	**NB**	0.60 (-0.07)	**0.66** (+0.09)	0.61 (+0.12)	0.22 (+0.22)	0.5 **(+0.46)**	0.64 (+0.36)	0.56 **(+0.49)**
	**GB**	**0.81** (-0.14)	**0.87** (+0.13)	**0.79** (+0.29)	**0.60** **(+0.60)**	**0.82** **(+0.82)**	0.68 **(+0.68)**	**0.74** **(+0.74)**
	**GCN**	**0.82** (-0.13)	**0.88** (+0.16)	**0.81** (+0.21)	**0.62** (+0.27)	**0.74** (+0.08)	**0.79** **(+0.6)**	**0.77** **(+0.46)**
	**MPNN**	0.78 (-0.16)	0.78 **(+0.28)**	0.86 **(+0.23)**	0.55 **(+0.55)**	0.69 **(+0.69)**	**0.78** **(+0.78)**	0.73 **(+0.73)**
	**ChemBERTa**	**0.82** (-0.14)	**0.80** (+0.18)	**0.87** (+0.06)	**0.62** (+0.12)	**0.83** (-0.17)	**0.71** **(+0.46)**	**0.76** **(+0.36)**
